# Non-oxidized bare copper nanoparticles with surface excess electrons in air

**DOI:** 10.1038/s41565-021-01070-4

**Published:** 2022-02-10

**Authors:** Kyungwha Chung, Joonho Bang, Athira Thacharon, Hyun Yong Song, Se Hwang Kang, Woo-Sung Jang, Neha Dhull, Dinesh Thapa, C. Muhammed Ajmal, Bumsub Song, Sung-Gyu Lee, Zhen Wang, Albina Jetybayeva, Seungbum Hong, Kyu Hyoung Lee, Eun Jin Cho, Seunghyun Baik, Sang Ho Oh, Young-Min Kim, Young Hee Lee, Seong-Gon Kim, Sung Wng Kim

**Affiliations:** 1 Department of Energy Science, Sungkyunkwan University, Suwon, Republic of Korea; 2 Center for Integrated Nanostructure Physics, Institute for Basic Science, Suwon, Republic of Korea; 3 Department of Physics and Astronomy and Center for Computational Sciences, Mississippi State University, Mississippi State, MS USA; 4 School of Mechanical Engineering, Sungkyunkwan University, Suwon, Republic of Korea; 5 Department of Materials Science and Engineering, KAIST, Daejeon, Republic of Korea; 6 Department of Materials Science and Engineering, Yonsei University, Seoul, Republic of Korea; 7 Department of Chemistry, Chung-Ang University, Seoul, Republic of Korea; 8 Present Address: Research Institute of Industrial Science and Technology, Pohang, Republic of Korea

**Keywords:** Nanoparticles, Nanoparticles

## Abstract

Copper (Cu) nanoparticles (NPs) have received extensive interest owing to their advantageous properties compared with their bulk counterparts. Although the natural oxidation of Cu NPs can be alleviated by passivating the surfaces with additional moieties, obtaining non-oxidized bare Cu NPs in air remains challenging. Here we report that bare Cu NPs with surface excess electrons retain their non-oxidized state over several months in ambient air. Cu NPs grown on an electride support with excellent electron transfer ability are encapsulated by the surface-accumulated excess electrons, exhibiting an ultralow work function of ~3.2 eV. Atomic-scale structural and chemical analyses confirm the absence of Cu oxide moiety at the outermost surface of air-exposed bare Cu NPs. Theoretical energetics clarify that the surface-accumulated excess electrons suppress the oxygen adsorption and consequently prohibit the infiltration of oxygen into the Cu lattice, provoking the endothermic reaction for oxidation process. Our results will further stimulate the practical use of metal NPs in versatile applications.

## Abstract (web summary)

A growth strategy based on surface-accumulated excess electrons enables the realization of non-oxidized bare copper nanoparticles stable under ambient conditions.

## Main

Copper (Cu) has an electron configuration of [Ar]3*d*^10^4*s*^1^ and tends to lose electrons, forming a rich variety of compounds, usually with oxidation states of +1 and +2; these chemical states formulate thermodynamically favourable cuprous oxide (Cu_2_O; standard enthalpy of formation Δ*H*°_f_ = −170 kJ mol^–1^) and cupric oxide (CuO, Δ*H*°_f_ = −156 kJ mol^–1^), respectively, in air^1^. This inevitable formation of Cu oxides under ambient conditions deteriorates the metallic properties of Cu, and the process is accelerated in Cu nanoparticles (NPs) with a large surface area, which have the potential to be a low-cost alternative to precious gold and silver analogues in various applications^2^. Thus, in the synthesis of Cu NPs, such as by wet chemical synthesis and solid-state thermal treatment (where stabilizers or supports are usually adopted to prevent the growth of particles over nanoscale dimensions), the artificial manipulation of surface structure via additional post-treatments is a mandatory process. One example is surface passivation with thiolate ligands to retard oxidation^3–5^. Another process, based on chemical etching^6^, thermal heat treatment^7^ and photoreduction^8^, is to eliminate the naturally formed Cu oxide moieties on the surfaces of Cu NPs. Although non-oxidized Cu NPs can be synthesized by the in situ reduction of Cu precursors in the form of composites with an epoxy matrix^9^, there is no practical way to autonomously prevent the oxidation of bare Cu NPs in air.

According to the Cabrera–Mott theory for oxidation of metals^10^, electrons from a metal migrate to the surface and bind with the adsorbed oxygen molecules, forming oxygen anions. Then, metal cations combine with the oxygen anions due to the surface dipole between them, and metal oxides form at the surface. This oxidation mechanism, thus, implies that it is possible to prevent the oxidation of a metal by preventing the release of electrons from metal atoms or by hindering the adsorption of oxygen. Indeed, the classic cathodic protection technique prevents metal corrosion by supplying additional electrons to realize a negatively charged state with excess electrons, which can suppress the ionization of metal into the cation^11,12^. Applying this strategy to metallic NPs can be a radical solution to the naturally occurring oxidation problem of Cu NPs without artificial manipulations of the surface structure. However, it is difficult to apply the cathodic protection technique for metal NPs in ambient conditions due to non-conducting air and the impractical targeting of each NP to supply currents. To realize bare Cu NPs having excess electrons for a negatively charged surface state, we conceived a rational one-step synthesis route that enables simultaneous growth and electron transfer, in which Cu NPs are grown on the surface of a support having an extraordinary electron-donating ability. Here we successfully demonstrate non-oxidized Cu NPs in air over several months, without a surface layer of Cu oxide even on the monoatomic scale, by employing inorganic electrides as electron-donating materials. Notably, no additional post-treatments or surface passivation processes are required.

Electrides, which are used as a support in the present synthesis of bare Cu NPs, are ionic crystals that exhibit exotic properties based on excess anionic electrons occupying a structural space of ~0.4 nm (refs. ^13–15^). The most distinct characteristic of electrides is a low work function^16^ that allows the effective transfer of anionic electrons to a material with a higher work function. It has been demonstrated that anionic electrons are transferred to chemically and physically adsorbed materials such as ruthenium particles and carbon nanotubes, showing enhanced catalytic and emission properties^17,18^. In this context, we grew Cu NPs on the surface of [Gd_2_C]^2+^·2e^−^ electride with an extremely low work function (~2.8 eV)^19,20^ and successfully obtained bare Cu NPs having excess electrons that are accumulated on the surface, exhibiting ultrahigh oxidation resistance under ambient conditions.

## Non-oxidized surface of bare Cu NPs

**Fig. 1:**
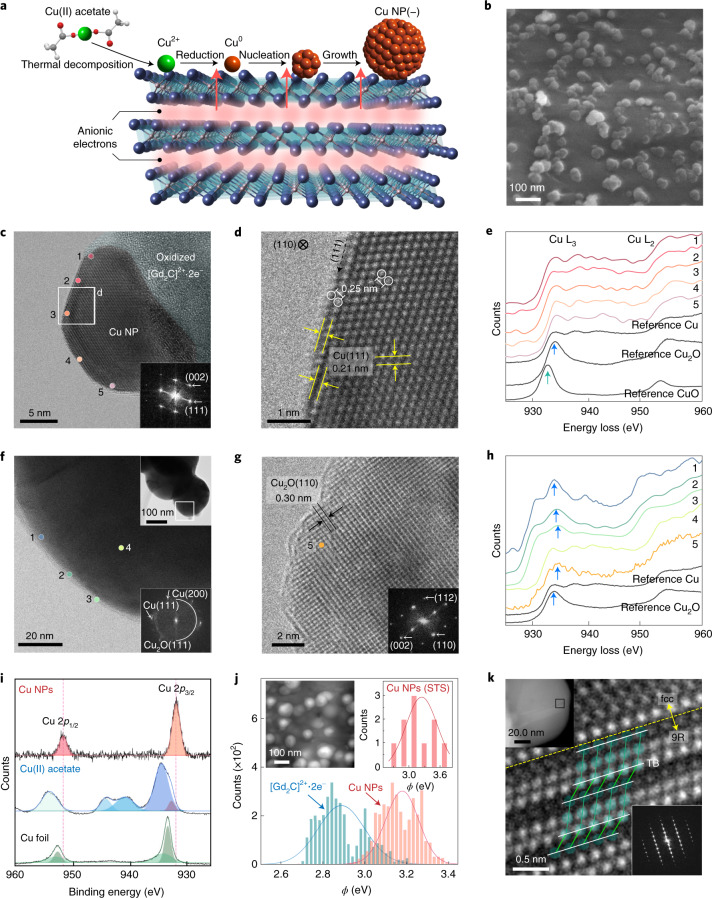
Negatively charged surface state of Cu NPs grown on a two-dimensional electride. **a**, Schematic of Cu NP growth on two-dimensional [Gd_2_C]^2+^·2e^−^ electride. **b**, SEM image of Cu NPs on the electride. **c**,**d**, HR-TEM image of the as-prepared Cu NPs on the oxidized electride (inset: fast Fourier transform (FFT) pattern) (**c**) and enlarged TEM image showing the atomic structure of fcc Cu(110) (**d**). **e**, EELS data for positions 1–5 at the surface in **c**. **f**, HR-TEM image of conventional Cu NPs prepared by the thermal reduction method (top inset: overall morphology; bottom inset: FFT pattern). **g**. Enlarged TEM image of a conventional Cu NP, showing that the NP was oxidized to Cu_2_O with an interplanar distance of 0.30 nm. **h**, EELS data for positions 1–3 at the surface and position 4 in the bulk (as shown in **f**) and for position 5 (as shown in **g**). Except for position 4, all the spectra show the white lines of Cu_2_O (blue arrows). **i**, XPS spectra of Cu NPs, Cu(ii) acetate and Cu foil. Two satellites (940.5 and 944.0 eV) for Cu(ii) acetate are the characteristics of Cu^2+^. **j**, Work-function (*ϕ*) values of Cu NPs and [Gd_2_C]^2+^·2e^−^ electride obtained by KPFM measurements. The peaks of the solid lines represent the average values (*ϕ*_av_) for Cu NPs (~3.2 eV; orange) and [Gd_2_C]^2+^·2e^−^ electride (~2.9 eV; blue) from all the measurements (solid bars). Inset: scanning tunnelling microscopy (STM) image of Cu NPs on [Gd_2_C]^2+^·2e^−^ electride (left) and *ϕ*_av_ of Cu NPs obtained by STS (~3.3 eV) (the set-point conditions are as follows: bias voltage *V* = 3 V; tunnelling current *I* = 500 pA) (right). **k**, HR-TEM image of the 9R phase in Cu NPs from the boxed area in the top inset. The twin boundary (TB; white lines) and atomic structures (green balls) correspond to the 9R phase. Bottom inset: FFT pattern of the 9R phase. Source data

Figure 1a illustrates the synthesis pathway of Cu NPs via spontaneous electron transfer from [Gd_2_C]^2+^·2e^−^ electride, producing well-dispersed Cu NPs on the surface of the electride (Fig. 1b). The as-prepared Cu NPs were analysed after exposing them to air for several minutes; this process led to the complete oxidation of the [Gd_2_C]^2+^·2e^−^ electride (Supplementary Fig. 1); no further transfer of excess anionic electrons occurred, making Cu NPs electrically isolated as similar to the contact-separation electrification process^21–23^, which can be seen in triboelectric nanogenerators^24^. A typical transmission electron microscopy (TEM) image of a Cu NP on the completely oxidized [Gd_2_C]^2+^·2e^−^ electride is shown in Fig. 1c. A high-resolution TEM (HR-TEM) image of the surface region clearly shows the face-centred cubic (fcc) structure with an interplanar distance of 0.21 nm for the {111} plane and an interatomic distance of 0.25 nm, which can be observed in the fcc Cu{110} plane (Fig. 1d and Supplementary Fig. 2). It is noteworthy that no surface oxidation occurred. Nevertheless, due to the ambiguity that a similar *d*-spacing value can be observed in the cubic Cu_2_O {200} plane, a chemical analysis was performed by electron energy loss spectroscopy (EELS) to verify the absence of Cu oxide moieties at the surface. It is apparent that all the EELS data obtained for the Cu NPs exhibit the same energy-loss near-edge structure of Cu L-edges as that of Cu metal without white lines (arrows) of Cu oxides^25,26^, demonstrating that the surfaces of Cu NPs are non-oxidized in air (Fig. 1e and Supplementary Fig. 3). In contrast, conventional Cu NPs (synthesized and commercialized Cu NPs) showed an oxidized surface of Cu_2_O after air exposure for 5 min, as expected (Fig. 1f–h and Supplementary Fig. 4). These observations emphasize that the present Cu NPs grown on the electride have strong resistance to oxidation.

## Negatively charged surface state of Cu NPs

Microscopic X-ray photoelectron spectroscopy (XPS) measurements show lower Cu 2*p*_3/2_ binding energy for Cu NPs (931.8 eV) than that for Cu foil (932.6 eV), which imply a higher electron density in Cu NPs (Fig. 1i). This negative shift suggests that the surface of Cu NPs has more excess electrons by the electron transfer from [Gd_2_C]^2+^·2e^−^ electride with a lower work function (~2.8 eV) than that of Cu metal (4.5 eV)^27^ (Supplementary Fig. 5). Thus, we can assume that when the ubiquitous formation of Cu nuclei and growth of Cu NPs occur, the anionic excess electrons of the electride are continuously transferred to the growing NPs. The transferred excess electrons are then accumulated at the surface of grown Cu NPs according to the Gauss theorem of electrostatics^28^. Direct experimental evidence for the surface state with excess electrons was obtained from work-function measurements using Kelvin probe force microscopy (KPFM) and scanning tunnelling spectroscopy (STS). Both histograms obtained from KPFM and field-emission resonance spectrum measured in the STS show substantially reduced work-function values (*ϕ*) for Cu NPs on [Gd_2_C]^2+^·2e^−^ (~3.2 eV on average), which are much lower than 4.5 eV for Cu metal (Fig. 1j and Supplementary Fig. 5)^29–31^. We also found that the structural instability of the fcc lattice of Cu NPs was induced by the transferred electrons, resulting in the local transition to the 9R Cu phase with an undulating threefold stacking fault-like structure^32^ (Fig. 1k and Supplementary Fig. 6). From first-principles calculations, it is clarified that the 9R Cu phase evolves due to the energetic instability of the fcc lattice with respect to the electronic structure, inducing the local phase transition to the 9R structure (Supplementary Fig. 6).

## Long-term air stability of non-oxidized bare Cu NPs

**Fig. 2:**
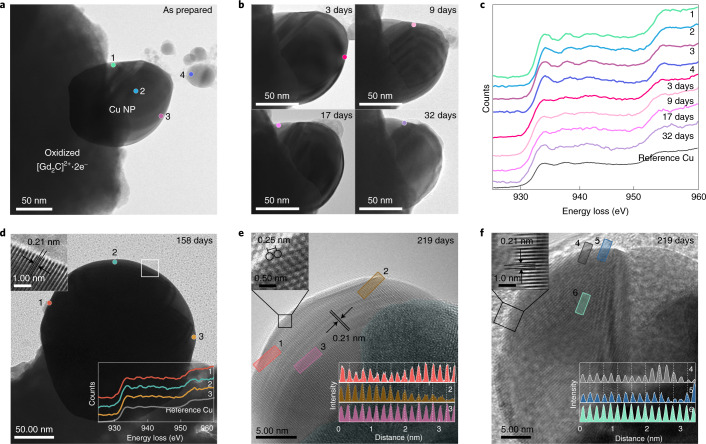
Non-oxidized surface of Cu NPs with a negatively charged state in air. **a**,**b**, TEM images of Cu NPs; as prepared (**a**) and as a function of exposure time in air (**b**). **c**, EELS data from positions 1–4 shown in **a** and the surface for each position shown in **b**. The separated NP from the oxidized electride (position 4) is also metallic Cu, indicating that excess electrons are transferred in the growth process of Cu NPs. **d**, Cu NP exposed to air for 158 days. The enlarged image (top inset) confirms the absence of a surface layer of Cu oxide and the interplanar distance of 0.21 nm of the Cu{111} plane. EELS data (bottom inset) also prove that the Cu NPs persist against oxidation. **e**, HR-TEM image of Cu NPs exposed to air for 219 days showing the interatomic distance of 0.25 nm for fcc Cu(110) (top inset). **f**, HR-TEM image of Cu NPs exposed to air for 219 days showing the interplanar distance of 0.21 nm for fcc Cu(111) (top inset). The invariable interplanar distance profiles (bottom insets) of the surface regions (positions 1, 2, 4 and 5) and bulk regions (positions 3 and 6) clearly indicate that Cu NPs survive without oxidation for more than seven months. Source data

Non-oxidized bare Cu NPs have been further examined for their oxidation resistance in air. Figure 2 shows the time evolution of the structural morphology and surface chemical state of the Cu NPs. The overall shape remains nearly identical without collapses of morphology in air for a month (Fig. 2a,b), demonstrating the extraordinary structural stability of these Cu NPs. Figure 2c shows the chemical state at the surfaces for each observation in Fig. 2a,b (marked by filled circles). It should be noted that all the surfaces are in the metallic state with no trace of Cu oxides, demonstrating the sustainable long-term stability of the non-oxidized Cu NPs in air. Remarkably, the metallic surface is preserved in air for several months (Fig. 2d–f). The surface of a Cu NP exposed to air for 158 days exhibits the energy-loss near-edge structure of metallic Cu (Fig. 2d, bottom inset) closely akin to that of all the displayed non-oxidized Cu NPs. The interplanar distance (Fig. 2d, top inset) is also identical to that of the as-prepared Cu NPs (Fig. 1d). Moreover, notwithstanding air exposure for 219 days, structural features such as the interplanar distance of 0.21 nm (Fig. 2e,f, bottom insets) and interatomic distance of 0.25 nm (Fig. 2e, top inset) are the same as those of the as-prepared, air-exposed Cu NPs for different periods of time (Fig. 1d and Supplementary Figs. 7 and 8). These observations strongly indicate that Cu NPs have strikingly strong and long-lasting oxidation resistance. In addition, the XPS spectrum of Cu NPs exposed to air for 100 days also shows the chemical state of Cu 2*p* with a lower binding energy than metallic Cu, implying that the surface state with a higher electron density is remarkably sustained (Supplementary Fig. 9). Thus, we conclude that Cu NPs grown on the electride are autonomously resistant to oxidation in air for several months.

## Atomic surface structure confirms the absence of monolayer oxide

**Fig. 3:**
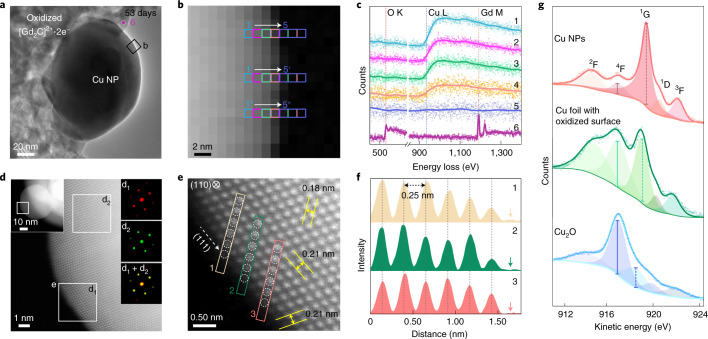
Surface analysis of air-exposed Cu NPs. **a**–**c**, EELS probing of the air-exposed Cu NP (53 days) shown in the TEM image (**a**), EELS mapping image with a nanometre-scale resolution through the acquisition of EELS signal from the three pixels marked as 1→5 (**b**) and the corresponding EELS data (**c**). Each spectrum is a sum of three different rows (*n* + *n*′ + *n*″). Spectrum 6 was obtained from oxidized [Gd_2_C]^2+^·2e^−^ electride (marked as 6 in **a**). **d**, ADF STEM image of the boxed region in the left inset of the air-exposed Cu NP (right insets: FFT patterns of the boxed regions). **e**, Atomic arrangement at the surface region (boxed regions in **d**). Interplanar distances (yellow arrows) of 0.18 and 0.21 nm are of the (002) and (1$\bar 1$1) planes, respectively. **f**, Interatomic distance profiles of six atoms along with the boxed arrays (indicated as 1–3) in **e** up to the vacuum (arrows). **g**, Cu L_3_M_45_M_45_ AES data of the Cu NPs (top), Cu foil with the oxidized surface of 1 nm thickness (middle) and Cu_2_O powder (bottom). Cu L_3_M_45_M_45_ AES data are fitted into five peaks. For Cu NPs, the kinetic energies and peak-area values for Cu NPs are consistent with a previous report^35^. The strong peak at 916.8 eV of Cu foil comes from the surface oxide, as revealed by the XPS data (Fig. 1i). In the Cu_2_O spectrum, the peak intensity at 916.8 eV is the strongest. Different intensity ratios between the peak at 916.8 eV (solid vertical line) and that between 918 and 919.2 eV (dashed vertical lines) for Cu NP with Cu foil indicate that 1-nm-thick surface oxide generates large discrepancy in the AES data, implying the non-oxidized surface state of Cu NPs. Source data

The detailed chemical and structural nature of the air-exposed surfaces of the charged Cu NPs was examined by scanning transmission electron microscopy (STEM)–EELS chemical mapping and atomic structure observations (Fig. 3a–f), proving that no Cu oxide forms at the outermost Cu atomic layer. At the surface of a Cu NP exposed to air for 53 days (Fig. 3a), the EELS data were collected from three consecutive pixels and accumulated (*n* + *n*′ + *n*″) (Fig. 3b and Supplementary Fig. 10). The EELS data from the three pixels in the region of Cu NPs (positions 1−4) to those of vacuum (position 5) are displayed in Fig. 3c. The EELS data from positions 1 to 4 consistently exhibit distinct signals at the Cu L-edge for Cu metal and no signal at the O K-edge, whereas the spectrum from position 5 shows no signal for both copper and oxygen, revealing that the surface is completely bare without oxide moieties. Position 6 shows definite peaks at the O K-edge and Gd M-edge, indicating the presence of Gd oxide moieties from the complete oxidation of the [Gd_2_C]^2+^·2e^−^ electride. We also confirmed no carbon adsorption at the surface (Supplementary Figs. 11 and 12), substantiating that the bare Cu NPs are non-oxidized. Furthermore, the surface atomic structure revealed by annular dark-field (ADF) STEM observations (Fig. 3d and Supplementary Fig. 13) also rules out the existence of Cu oxides—even monolayer oxide—at the surface of air-exposed Cu NPs. The insets in Fig. 3d show identical local diffraction patterns (d_1_ + d_2_; right-bottom inset) from the surface (d_1_; right-top inset) and the bulk (d_2_; right-middle inset), implying no oxidation at the surface area. From the high-resolution ADF STEM observations, which avoid the Fresnel fringes at the surfaces appearing in the HR-TEM images (Fig. 2e and Supplementary Fig. 8), the atomic arrangements at the outermost surface are clearly resolved, and a perfect regularity in interatomic and interplanar distances appears (Fig. 3e,f). This periodic arrangement up to the outermost atomic layer with an interatomic distance of 0.25 nm in the [1$\bar 1$0] direction strongly indicates that no Cu oxide forms, in contrast to the elongated interatomic distance (0.31 nm) between Cu atoms of bulk and monolayer Cu oxide^33^. This interatomic distance change in the metal atoms is a well-known feature in the surface study of oxidized metals, as observed at the initial stage of oxidation, such as the oxygen-adsorbed surface of silver NPs^34^. Thus, the constant atomic arrangement of Cu NPs with a periodicity of 0.25 nm up to the outermost surface concludes that Cu oxides do not form at the surface, even in the monolayer. This astonishing non-oxidized surface of bare Cu NPs in air is also confirmed by Auger electron spectroscopy (AES). Considering the theoretical and experimental studies on the AES data of Cu metal^35,36^, we analysed the AES data of our non-oxidized Cu NPs and compared with those of Cu metal foil having 1-nm-thick Cu_2_O (Fig. 3g, middle, and Supplementary Fig. 14), Cu_2_O powder (Fig. 3g, bottom) and Cu metal disc with monolayer Cu_2_O (ref. ^37^) (Supplementary Fig. 15). The L_3_M_45_M_45_ spectrum of our Cu NPs is well fitted by using the parameters for each state term^35^ of the Cu metal (Methods and Supplementary Fig. 15). Furthermore, from the clear difference in the transition probability for the state terms between our Cu NPs and three references having the Cu_2_O surface, it is evident that our Cu NPs have no oxidized layer of Cu_2_O at the surface in the bare form. Therefore, from high-resolution ADF STEM observations and AES measurements, the surface oxidation of the present Cu NPs is completely excluded.

## Surface-accumulated excess electrons of bare Cu NPs

Finally, using first-principles calculations (Methods), we explain how bare Cu NPs are not oxidized in air, validating our experimental results. First, we identified that the transferred excess electrons are accumulated at the surface, where the number of excess electrons on the surface Cu atoms is estimated by matching the calculated work-function value (3.25 eV) from theoretical models having different excess electrons to the average of the measured values of non-oxidized bare Cu NPs by KPFM (~3.2 eV) and STS (~3.3 eV) measurements (Fig. 1j). It was revealed that the average excess charges per surface Cu atom (*Q*) is −2.5*e* (*e* is the elemental charge), which is also verified by rigid-band approximation (Methods and Supplementary Fig. 16). This exceptionally large number comes from the surface accumulation of excess electrons transferred to the Cu clusters or NPs during the initial stage of growth process, in which a part of excess electrons is probably consumed for the local transition to the 9R phase in the bulk (Fig. 1k), and the remainder is expelled to the surface, leading to the negatively charged surface state. Next, we evaluated the energetics of the oxidation process of Cu NPs using a flat surface (FS) and a step edge (SE) of Cu lattices for both neutral (*Q* = 0*e*) and charged (*Q* = −2.5*e*) models, considering the morphology effect of Cu NPs^38^.

## Endothermic oxidation reaction of negatively charged Cu surface

**Fig. 4:**
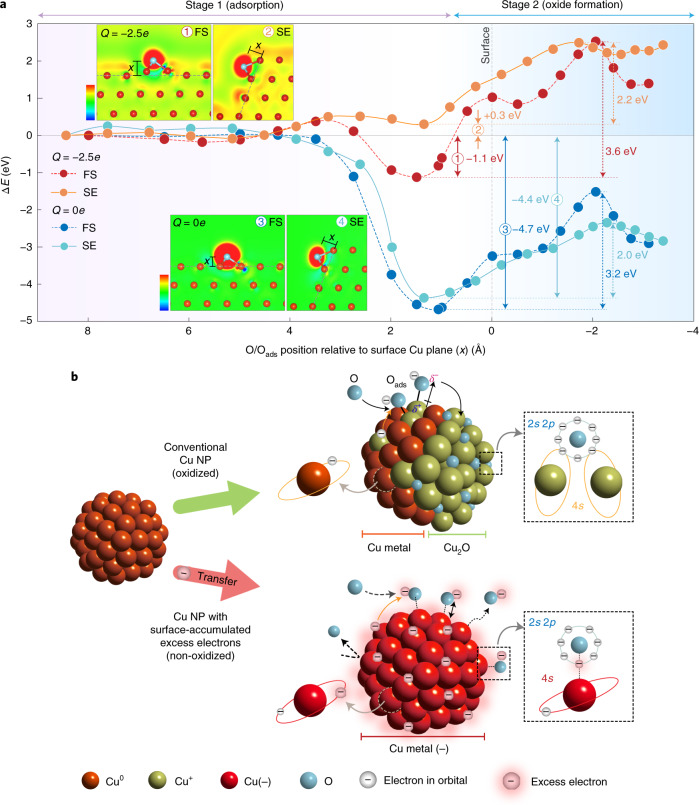
Theoretical analysis and model of oxidation-resistant Cu NPs. **a**, Relative energy profiles of the adsorption of O atom onto neutral (*Q* = 0*e*) (blue circle, FS; cyan circle, SE) and charged (*Q* = −2.5*e*) fcc Cu(111) facets (red circle, FS; orange circle, SE). The insets show the plots of excess electron density for O-adsorbed FS and SE of Cu(111) facets in neutral (*Q* = 0*e*) and charged (*Q* = −2.5*e*) states. The blue (red) colour represents the region of electron depletion (augmentation) with reference to neutral systems (scale bars represent the range from −0.037 to 0.037*e* Å^–3^ and from −0.057 to 0.057*e* Å^–3^ for FS and SE of Cu, respectively). **b**, Schematic of the model for the oxidation process of conventional Cu NPs (top) and non-oxidized Cu NPs with surface-accumulated excess electrons (bottom). *δ*^+^ and *δ*^–^ correspond to the amount of electron loss in Cu and electron gain in O, respectively. Source data

Figure 4a shows the relative energy profiles of the central processes in the formation of Cu oxides: O atom is chemically adsorbed (O_ads_) on the Cu atom at the surface (stage 1, adsorption) and O_ads_ infiltrates into the lattice to form an oxide (stage 2, oxide formation) (Supplementary Fig. 17 shows configurations of the detailed steps). Most importantly, we note that the endothermic process on the negatively charged surfaces in stage 2 directly proves no formation of Cu oxides at the surface as well as in the bulk, strongly supporting the experimental observations for the absence of monoatomic Cu oxide moieties at the surface. The unprecedented stability of Cu NPs in air is exclusively attributed to the surface-accumulated excess electrons, which dominate the energetics of the adsorption and oxide formation processes. When O atom adsorbs on the neutral Cu surface, O_ads_ is found at the hollow site of the Cu surface, with adsorption energies ranging from −4.7 eV (FS) to −4.4 eV (SE) (indicated by the blue and cyan solid arrows, respectively, in Fig. 4a), which are in good agreement with the well-defined value of −4.5 eV in the oxidation process of conventional Cu metal^39,40^. The O_ads_ does not immediately lead to oxide formation at the surface Cu layers, as it would require overcoming the activation energies of 3.2 eV (FS) or 2.0 eV (SE). The much smaller activation energy for infiltration at the SE is consistent with the previous observation in conventional Cu thin films, where the edges of atomic steps are most vulnerable to oxidation^33^. However, the overall reaction is exothermic, and the persistent presence of O_ads_ threatens the breach of the surface Cu layers when exposed to ambient conditions. Unlike the stabilized O_ads_ in the conventional oxidation process, the negatively charged surface makes O_ads_ much more unstable, with adsorption energies of −1.1 eV (FS) and +0.3 eV (SE) (indicated by the red and orange solid arrows, respectively, in Fig. 4a). This leads to a remarkable difference in the stability of O_ads_, as the energy gain from adsorption is reduced by as much as 3.6 eV (FS) and 4.7 eV (SE) in the charged system compared with those of the neutral system. This relatively unstable bonding between O_ads_ and charged Cu surfaces originates from the large number of surface-accumulated excess electrons, most of which remain at the surface even after transferring electrons to O atoms. These coexisting negative charges lead to Coulomb repulsion between negatively charged Cu surface and oxygen anions, strongly suppressing the adsorption of oxygen anions on the Cu surface and consequently prohibiting the infiltration of O_ads_ into the bulk lattice.

The insets of Fig. 4a show the distribution of the excess electrons of the Cu surfaces, where the negatively charged surface states are clearly distinguished from the surface states of the neutral system. In addition, the activation energies for the infiltration of O_ads_ into the Cu lattice increase from 3.2 to 3.6 eV (FS) and from 2.0 to 2.2 eV (SE) for neutral and negatively charged systems, respectively. It should be noted that the increase in adsorption energy by 3.6–4.7 eV in the negatively charged system predominates the infiltration process with a relatively small energy difference of 0.2–0.4 eV, indicating that the extraordinary oxidation resistance of the charged Cu NPs can be attributed to the severe suppression of oxygen adsorption on the surface. Therefore, in contrast to the general oxidation process through the dipole formation between Cu cations and oxygen anions, a substantial amount of energy is needed for the oxygen anions to be chemisorbed and to pass through the negatively charged outermost Cu layer, developing the endothermic reaction for oxide formation in Cu NPs with surface-accumulated excess electrons. The schematic of whole process is illustrated in Fig. 4b, highlighting the surface-accumulated excess electrons working as a natural passivating layer for oxidation.

## Conclusion

In summary, we developed non-oxidized bare Cu NPs in air by realizing a negatively charged surface state mimicking the classic cathodic protection^11,12^ at the nanoscale. Using the present methodology, not only Cu NPs but also silver NPs maintain non-oxidized surfaces under ambient conditions (Supplementary Fig. 18). We also synthesized non-oxidized bare Cu NPs by solid-state thermal treatment utilizing different two-dimensional electrides (Supplementary Fig. 19) as well as the wet chemical synthesis for mass production (Methods and Supplementary Fig. 20), providing a universal and radical solution to overcome the fundamental obstacle—surface oxidation—of metal nanoparticles for practical industrial applications. From the successful synthesis of non-oxidized bare Cu NPs by a solution process without using a surfactant or ligand, it is highlighted that the direct transfer of excess electrons to Cu NPs ensures their oxidation resistance in the mass-produced bare form, as confirmed by the surface atomic arrangement of fcc Cu in air-exposed Cu NPs after 15 and 31 days in air (Supplementary Fig. 21). In particular, the mass-produced bare Cu NPs also exhibited a non-oxidized surface state in deionized water for 60 min, demonstrating strong chemical stability under active circumstances. Enhanced performances of the non-oxidized Cu NPs in electrocatalysis and electrodes (Supplementary Figs. 19 and 21), benefitted from the excess electrons transferred from the electrides, support the practical utility of our non-oxidized bare Cu NPs. The present findings will initiate a new phase for metal NPs that can replace the noble gold and silver NPs in versatile applications, where a combined system of metal NPs and electrides will be of special interest as a potential platform for catalytic reactions promoted by an efficient electron transfer ability.

## Methods

### Synthesis of [Gd_2_C]^2+^·2e^−^ electride

All the synthesis processes were carried out in glove boxes filled with high-purity argon gas (Ar, 99.999%) to minimize the oxidation of raw materials. Polycrystalline [Gd_2_C]^2+^·2e^−^ electride is prepared by the arc-melting method using Gd metal pieces (Alfa Aesar, 99.9%) and graphite at a molar ratio of 2:1. To obtain high-quality single-phase [Gd_2_C]^2+^∙2e^−^ and ensure homogeneity, we repeated the cooling and re-melting process three times. After the melting process, the polycrystalline [Gd_2_C]^2+^∙2e^−^ ingot was moved into the glove box to grind the oxidized surface. Then, the fresh ingot was pulverized into a fine powder for producing Cu NPs.

### Preparation of Cu NPs

Polycrystalline [Gd_2_C]^2+^·2e^−^ powder (0.500 g) and dried Cu(ii) acetate (0.015 g) were mixed with *n*-heptane in an agate mortar in an argon atmosphere and transferred to a quartz tube for heating at 150 °C under a dynamic vacuum for 24 h. Conventional Cu NPs without a capping agent were prepared as a reference sample by the thermal reduction of Cu(ii) formate complex (CuF) with 2-amino-2-methyl-1-propanol (AMP)^41^. A CuF–AMP complex was obtained by mixing anhydrous CuF and AMP at a molar ratio of 1:2. The mixture was dispersed in methanol and heated to 180 °C for 4 h in a vacuum oven. Finally, Cu NPs were collected as the final product by drying in a vacuum. Commercial Cu NPs (diameter, ~40 nm; capped with polyvinylpyrrolidone (PVP)) prepared by the electric explosion method^42^ are used (US Research Nanomaterials). For demonstrating the practical applications of negatively charged Cu NPs, we also developed a simple and efficient method for preparing the negatively charged Cu NPs via a wet chemical process that enables the complete separation of Cu NPs from the electride. Cu precursors (anhydrous CuCl_2_; 0.5 mmol) were reduced by excess electrons of [Gd_2_C]^2+^·2e^−^ electride chips (1.0 mmol) in anhydrous 1-hexanol (5 ml) at 80 °C under mild stirring, and then [Gd_2_C]^2+^·2e^−^ electride chips were removed by using a magnet. This method uses no additional stabilizing agent, which is essential for reducing the surface energy of nanoparticles in conventional synthesis methods of metal NPs. Furthermore, the product was easily separated using a magnet, obviating the need for post-treatment. The collected Cu NPs were washed by centrifugation and tip sonication (Supplementary Fig. 20).

### Characterization of Cu NPs

#### TEM and EELS

All the samples were dispersed in a non-polar solvent (*n*-heptane) and applied to a QUANTIFOIL 300 mesh gold holey carbon grid (Quantifoil Micro Tools) in an argon-filled glove box and then further dried in a vacuum chamber. For Cu NPs with [Gd_2_C]^2+^·2e^−^ electride, before TEM observations, as-prepared Cu NPs with [Gd_2_C]^2+^·2e^−^ electride samples were exposed to ambient air for 5 min to trigger the oxidation of [Gd_2_C]^2+^·2e^−^ electride for excluding any possible charge transfer between Cu and electride support. To investigate the oxidation behaviour of Cu NPs, TEM grids prepared as above were transferred into uncapped glass vials and exposed to ambient air at room temperature during designated time periods. HR-TEM, energy-dispersive X-ray spectroscopy and STEM–EELS measurements were performed using a Cs-corrected JEM-ARM200F probe (JEOL) operated at acceleration voltages of 200 and 300 kV. For EELS mapping, the sample drift during acquisition was compensated by tracking the position of the reference atom assigned at the beginning of the acquisition. EELS measurements were carried out in the STEM mode using the same microscope equipped with a Gatan imaging filter (GIF) detector (Model 965, GIF Quantum ER). The spectrometer was set to an energy dispersion of 0.2 eV per channel, and the energy resolution at the zero-loss peak is 0.9 eV.

#### SEM and XRD

Scanning electron microscopy (SEM) images of Cu NPs were obtained using a field-emission scanning electron microscope (JSM-7600F, JEOL). The crystal structure of [Gd_2_C]^2+^·2e^−^ electride with Cu NPs was investigated by X-ray diffraction (XRD) using an X-ray diffractometer (Rigaku Smartlab) with monochromatic Cu Kα1 line (8.04 keV). The XRD patterns of the as-prepared Cu NPs on [Gd_2_C]^2+^·2e^−^ were measured in a plastic dome-type stage filled with argon gas to avoid oxidation during measurements. Time-dependent XRD patterns were obtained by repeating the measurement with the same samples after exposure to ambient air at room temperature.

#### XPS/UPS and AES

XPS of Cu 2*p*, Cu L_3_M_45_M_45_ AES and ultraviolet photoelectron spectroscopy (UPS) were measured using an R4000 spectrometer (VG Scienta) with Al Kα X-ray source (1,486.7 eV) and He Iα (21.22 eV). The XPS and AES data were calibrated using the Au 4*f* peak at 84.0 eV as the reference. Cu L_3_M_45_M_45_ AES was obtained by using the Cu NPs synthesized by a wet chemical process to avoid the background disturbance of Gd M_45_N_45_N_45_ Auger line arising in the binding energy of 565–680 eV. AES was modelled by a symmetric mixed Gaussian–Lorentzian function^39^.

#### KPFM

The surface work function of the samples deposited on a Au-coated Si substrate was measured by an atomic force microscope (MFP-3D AFM, Asylum Research) equipped with a sealed electrochemistry cell filled with argon gas. The KPFM experiments were conducted using Ti/Ir-coated Si probe (ASYELEC.01-R2, Asylum Research) with a force constant of 2.8 N m^–1^ and a resonance frequency of 75 kHz. The work function of this probe was calibrated using a highly ordered pyrolytic graphite (HOPG) reference sample with a well-defined work function (4.6 eV)^43^. During the KPFM scanning process, the scan rate and set point were 0.8 Hz and 0.5 V, respectively. Furthermore, an a.c. voltage *V*_a.c._ = 1 V was applied to the tip, and the tip was lifted up 30 nm from the sample surface. The other scanning parameters were also optimized for obtaining high-quality images. The similarity between the measured work-function value (2.88 eV) and the calculated one (2.78 eV) for the [Gd_2_C]^2+^·2e^−^ electride ensures the reliability of the extremely low work function of negatively charged Cu NPs.

#### STM/STS

STM/STS measurements were performed in an ultrahigh vacuum chamber with a base pressure of ~1.0 × 10^–10^ torr at room temperature using a commercial STM instrument (VT SPM, Omicron). An electrochemically etched tungsten tip was used after removing oxides by electron bombardment in the ultrahigh vacuum chamber. The tips were calibrated by measuring the reference spectra on HOPG to avoid tip artifacts. A tunnelling bias was applied to the sample. Further, *z*–*V* spectroscopy was done by sweeping the tunnelling bias (*V*) and monitoring the tip height (*z*) when the feedback loop is on.

#### Theoretical calculation

All the calculations are based on first-principles density functional theory as implemented in the Vienna ab initio simulation package^44^ with the projector augmented-wave method; a generalized gradient approximation in the Perdew–Burke–Ernzerhof form is adopted for the exchange–correlation functional^45^. The electron wavefunctions were expanded in a plane-wave basis set with a cutoff energy of 500 eV, and the Brillouin-zone integration for the slabs was performed using a 5 × 5 × 1 Monkhorst–Pack *k*-point grid^46^. The climbing-image nudged elastic band method^47^ was used to calculate the activation energy of oxidation, and all the atoms are fully relaxed until the residual forces on each atom are less than 0.01 eV Å^–1^. The Cu substrate was represented by slabs of six layers with the theoretical equilibrium lattice constant. A vacuum length of 25 Å was used, and the bottom two layers of the slab were fixed in their bulk positions. For surface energy calculations, the fcc Cu(111), Cu(100) and Cu(110) systems consist of (4 × 4), (3$\sqrt 2$ × 3$\sqrt 2$) and (3$\sqrt 2$ × 4$\sqrt 2$) surface unit cells with four layers, resulting in supercells of 64, 72 and 96 Cu atoms, respectively. The spurious electrostatic energy associated with long-range Coulomb interactions between the supercells were corrected using monopole and multipole terms by the schemes implemented in the Vienna ab initio simulation package^48,49^. The charge difference density of the Cu NP charged at *Q* excess charges per surface Cu atom or neutral (*Q* = 0*e*) is computed as follows: Δ*ρ*^(*Q*)^ = *ρ*^(*Q*)^[Cu + O] – (*ρ*^(0)^[Cu] + *ρ*^(0)^[O]), where *ρ*^(*Q*)^[Cu + O] is the charge density of Cu NP with O_ads_ on its surface and *ρ*^(0)^[Cu] and *ρ*^(0)^[O] are the charge densities of the neutral Cu substrate and isolated O atom at the position of O_ads_, respectively. The rigid-band approximation was adopted to verify the calculational results for correlation between the excess electrons and work functions. The work-function change of the Cu(111) slab after adopting the excess electrons can be defined as Δ*ϕ* = *ϕ*_excess_ – (*ϕ*_neutral_ – Δ*E*_F_), where *ϕ*_excess_ and *ϕ*_neutral_ are the work functions of the excess and neutral Cu slabs, respectively, and Δ*E*_F_ is the variation in the Fermi level of the system due to excess electrons. The Δ*E*_F_ values were extracted using the density of states of the neutral Cu(111) slab (Supplementary Fig. 16d–f).

#### Preparation and characterization of Cu NP inks and electrodes

For the preparation of conductive Cu NP inks, powder of bare Cu NPs synthesized by the wet chemical method are added into the dispersion of PVP (molecular weight, 40,000) in isopropyl alcohol. PVP acts as an adhesive at the interface between the Cu NPs and glass substrate. The mass ratio for the Cu, adhesive and solvent is fixed to 5.0:0.6:94.4. The mixture is then tip sonicated for 15 min (560 W) to ensure homogeneity. A powder of PVP-capped commercial Cu NPs is used as the control material in the same procedure. Further, 40 μl of the prepared conductive Cu NP inks were dropped onto a predefined mould (20.00 × 5.00 × 0.05 mm^3^) on a glass substrate and aged for 12 h at room temperature. A curing process was conducted at 250 °C for 60 min in an inert atmosphere. The electrical conductivities of the electrodes were measured using a four-point-probe in-line method with a current source (Keithley 6221) and a nanovoltmeter (Keithley 2182A)^50^. The distance between the probes is 1 mm. The volume resistivity (*ρ*) was then calculated using the formula *ρ* = *C*(*V*/*I*)*t*, where *C* is a geometric correction factor, *V* is the measured voltage, *I* is the supplied current and *t* is the thickness of the electrode determined using a stylus profilometer (Alpha-Step D-100). The geometric correction factor (*C* = 3.575) of the volume resistivity is applied as discussed elsewhere^51^.

## Online content

Any methods, additional references, Nature Research reporting summaries, source data, extended data, supplementary information, acknowledgements, peer review information; details of author contributions and competing interests; and statements of data and code availability are available at 10.1038/s41565-021-01070-4.

## Supplementary information

Supplementary Information Supplementary Figs. 1–21 and references.

## Data Availability

Source data are provided with this paper. Any additional data are available from the corresponding authors upon reasonable request.

## Source data

Source Data Fig. 1 Source data of EELS for our Cu NPs and reference materials (Fig. 1e), source data for conventional Cu NPs (Fig. 1h), source data of XPS of the samples and the fitted peaks (Fig. 1i) and histogram of work function measured by KPFM and STS (Fig. 1j).
Source Data Fig. 2 Source data of EELS for our Cu NPs (Fig. 2c,d) and source data of interplanar distance profiles (Fig. 2e,f, insets).
Source Data Fig. 3 Source data of EELS mapping data without subtracting the background (Fig. 3c); source data for interatomic distance profiles (Fig. 3f); source data for X-ray-excited AES data of three samples and the fitted peaks, background and envelope (Fig. 3g).
Source Data Fig. 4 Source data for the relative energy profiles of oxygen adsorption (Fig. 4a).
