# Supplementary material for: Non-oxidized bare copper nanoparticles with surface excess electrons in air
Source: Nat Nanotechnol. 2022 Feb 10;17(3):285–91. doi: 10.1038/s41565-021-01070-4 (PMC8930766; doi:10.1038/s41565-021-01070-4)
Supplement: Supplementary file 1 — Supplementary Figs. 1–21 and references. [file 41565_2021_1070_MOESM1_ESM.pdf]

nature portfolio

https://doi.org/10.1038/s41565-021-01070-4

Supplementary information

Non-oxidized bare copper nanoparticles with surface excess electrons in air

In the format provided by the authors and unedited

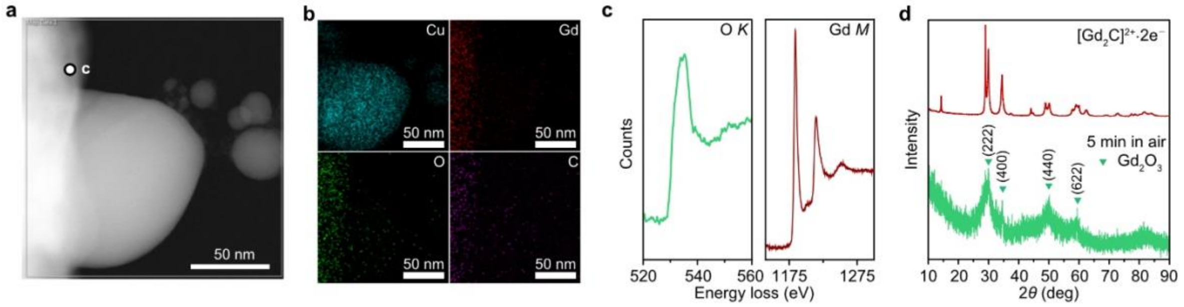

Supplementary Fig. 1 | Elemental and structural analyses of the $[Gd_2C]^{2+}{\cdot}2e^-$ electride with as-prepared Cu NPs after air exposure for 5 min. a–b, Energy-dispersive X-ray (EDX) analysis was conducted on Cu NPs grown on $[Gd_2C]^{2+}{\cdot}2e^-$ electride after the complete oxidation of $[Gd_2C]^{2+}{\cdot}2e^-$ electride in air as shown in STEM-HAADF image (**a**). Elemental mapping for copper, gadolinium, oxygen, and carbon (**b**). **c**, EEL spectra of oxygen *K* edge (left) and Gd *M* edge (right) obtained from the air-exposed $[Gd_2C]^{2+}{\cdot}2e^-$ electride marked with white circle in **a**. **d**, Powder XRD patterns of as-prepared (top) and air-exposed (bottom) $[Gd_2C]^{2+}{\cdot}2e^-$ electride. The $[Gd_2C]^{2+}{\cdot}2e^-$ electride was completely oxidized by exposure in air for 5 minutes losing all anionic electrons upon the reaction with oxygens and water molecules in air. This oxidation process of $[Gd_2C]^{2+}{\cdot}2e^-$ electride obviates further transfer of the excess electrons to Cu NPs.

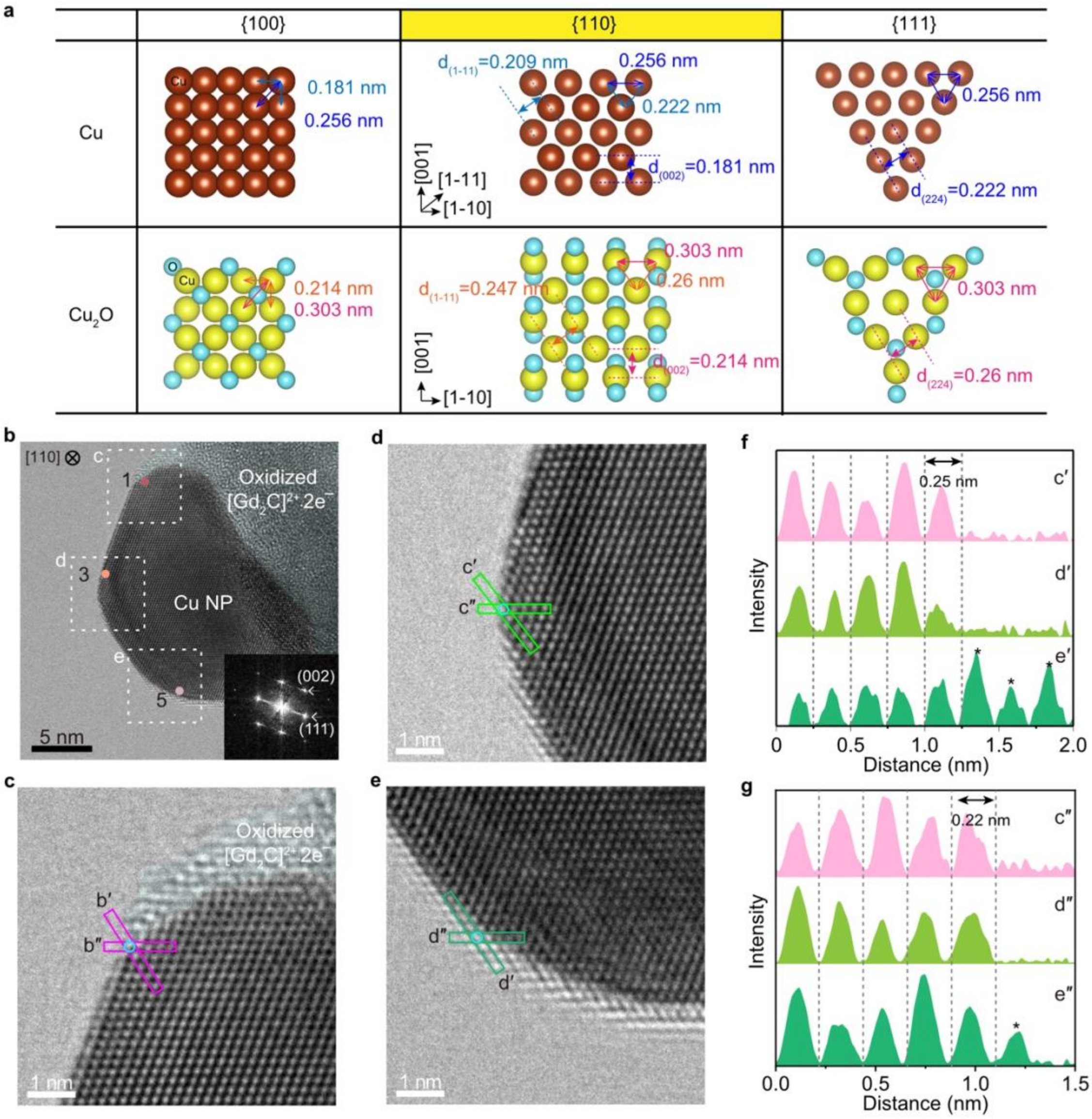

Supplementary Fig. 2 | Crystal structures of copper. a, Comparison for structural configurations of {100}, {110} and {111} planes of fcc Cu (space group: Fm$\overline{3}$m) and Cu_2O (space group: Pn$\overline{3}$m). Interplanar and interatomic distances are indicated for each structure. **b**–**e**, TEM image of the Cu NP presented in Fig. 1c of main text (**b**) and enlarged HR-TEM images of the corner area marked with white dashed boxes (**c**, **d**, and **e**). Interatomic distance profiles of the outermost five atoms along <110> direction (b', c', and d') (**f**) and along <111> direction ((b", c", and d") (**g**). Asterisk symbols indicate signal from Fresnel fringes.

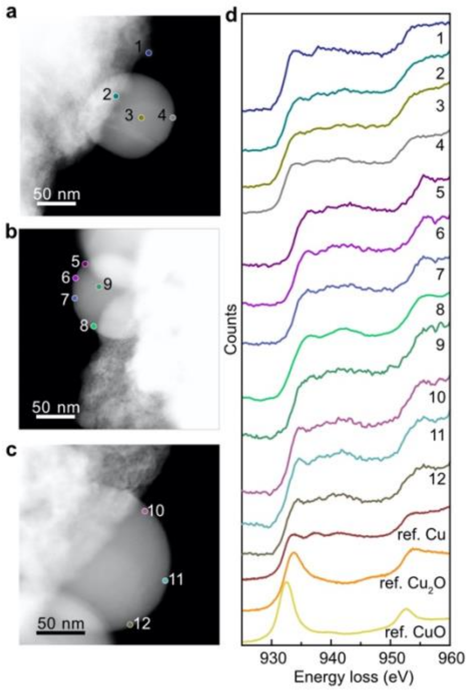

Supplementary Fig. 3 | EELS of as-prepared Cu NPs grown on the electride. a–**d**, TEM images (**a**–**c**) and EELS measurements (**d**) of as-prepared Cu NPs grown on the $[Gd_2C]^{2+}\cdot 2e^-$ electride (after exposing to air for several minutes). Cu NPs exhibit the energy-loss near-edge structure (ELNES) of Cu *L* edges as that of Cu metal without white lines of copper oxides, confirming that the surfaces of the Cu NPs are non-oxidized. In all spectra, no white lines of copper oxides are observed.

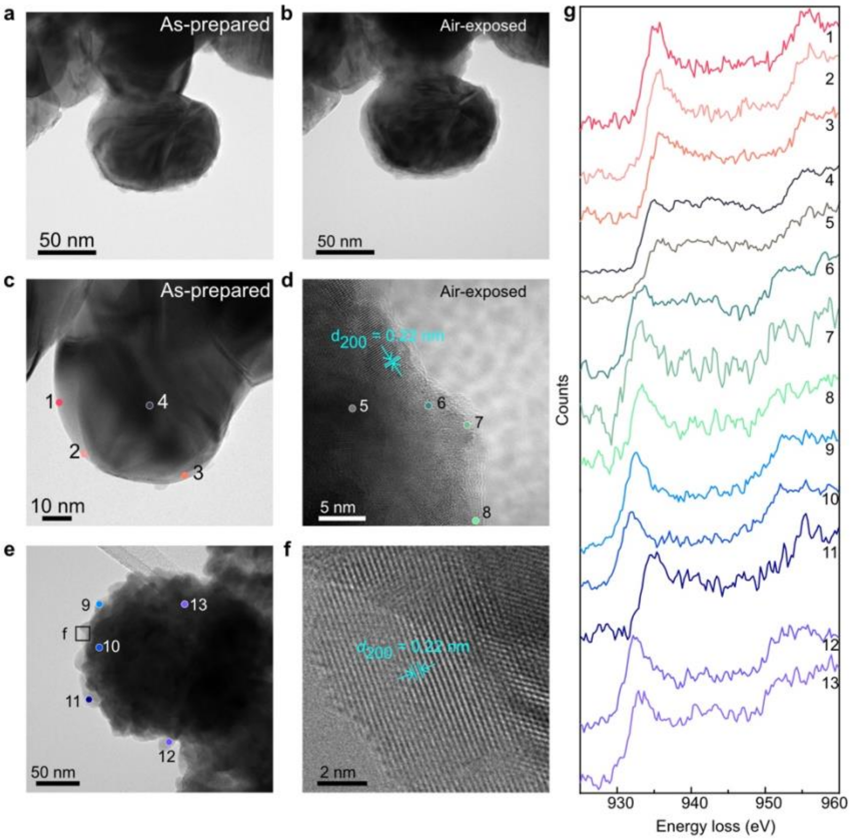

Supplementary Fig. 4 | EELS of conventional Cu NPs. a–**f**, TEM images and EELS measurements of conventional Cu NPs. TEM images of as-prepared (**a**,**c**) and air-exposed (for 5 minutes) (**b**,**d**) Cu NPs synthesized by thermal reduction of CuF-AMP complex, and Cu NPs prepared by electric explosion method (as-prepared) (**e**–**f**) show that conventional Cu NPs are severely oxidized after air exposure. **g**, EELS of Cu L edges on the surface of the conventional Cu NPs obtained from the circles in (**a**–**f**). For Cu NPs prepared from CuF-AMP complex, EEL spectra from the surface (1–3) clearly show the white line of Cu_2O, while the bulk (4 and 5) exhibited ELNES of Cu metal. In the case of commercial Cu NPs (9–13), the white lines are observed in energy loss spectra originated from Cu_2O even at the bulk (10 and 13). Interplanar distances of 0.22 nm in **d** and **f** also indicate that of Cu_2O {200} planes.

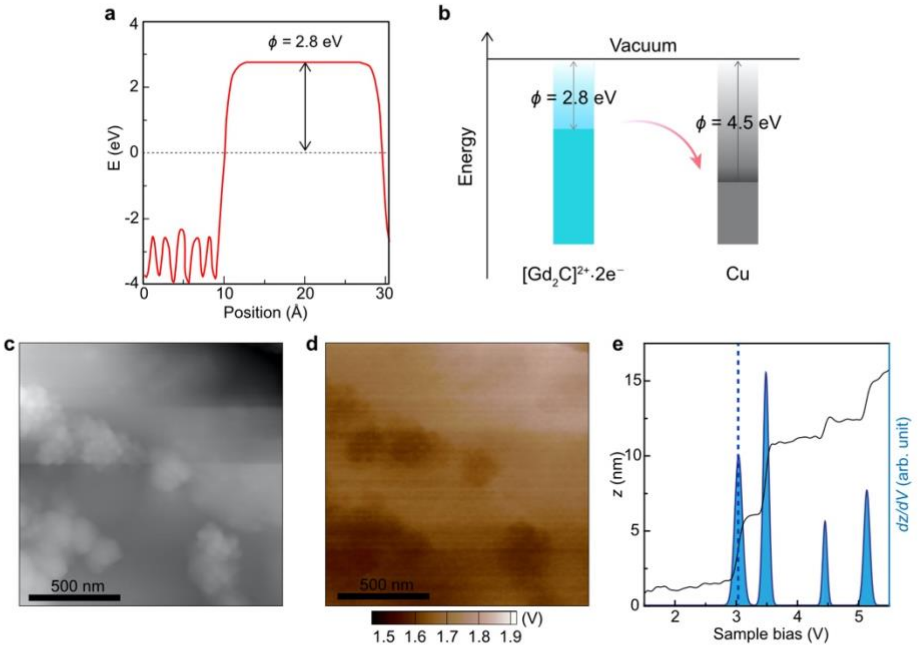

Supplementary Fig. 5 | Electron transfer from $[Gd_2C]^{2+}·2e^{-}$ electride to Cu NPs. a, Calculated work function ϕ (2.8 eV) for the (110) surface of $[Gd_2C]^{2+}·2e^{-}$ electride. **b**, Schematic illustration showing the electron transfer from the electride to Cu NPs. After the formation of Cu nuclei, a large difference in work function between two materials causes the transfer of excess anionic electrons of the electride to Cu metal. **c**–**e**, KPFM and STS measurement results. Topography (**c**) and contact potential difference (CPD) mapping (**d**) images obtained by KPFM for the as-prepared CuNPs on $[Gd_2C]^{2+}·2e^{-}$ electride. Field emission resonance (FER) spectrum (**e**) was obtained to measure ϕ of Cu NPs using STS by differentiating tip-to-sample distance (z) with bias voltage (V) required to maintain the constant current, in which the first FER peak position corresponds to the work function (black line: z-V curve, blue line: dz/dV spectrum)[29–31]. In this spectrum, the work function is measured to be 3.0 eV (dashed line).

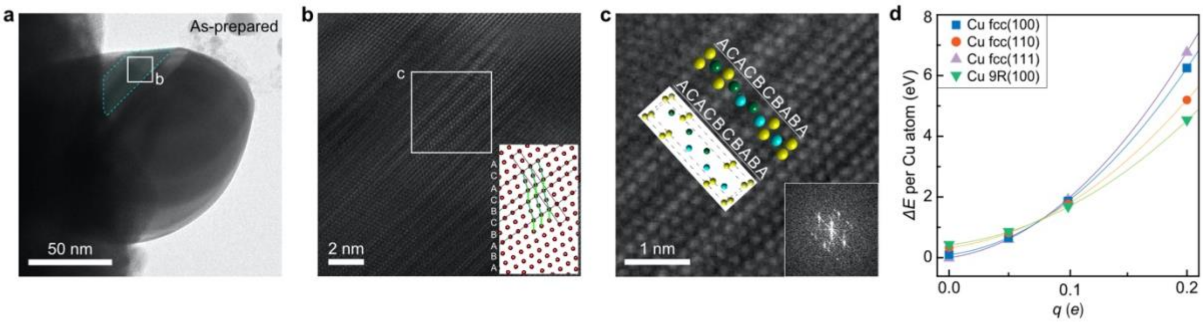

Supplementary Fig. 6 | 9R structure in the Cu NP grown on $[Gd_2C]^{2+}\cdot 2e^-$ electride. a–c, 9R structure in the Cu NP grown on the electride. **a**, TEM image of the as-prepared Cu NPs displayed in Fig. 2 shows a twin-rich region marked with a dotted blue line. **b**, Enlarged HR-TEM image of boxed region in **a**. Inset: (100) plane of 9R Cu showing undulated atomic arrange with dense twin boundaries. **c**, Enlarged HR-TEM image of boxed region in **b**, matching well with the 9R Cu lattice (inset: FFT diffraction pattern). This 9R structure was observed in relatively large Cu NPs (> ~90 nm). The evolution of the 9R phase, which is a type of lattice defects, occurs when the transfer of excess electrons has sufficiently proceeded in the growing NPs. **c**, *Ab initio* calculation on the change in thermodynamic stability of different Cu facets with the increasing number of excess electrons per a Cu atom (q) implies that (100) plane of 9R Cu becomes the most stable surface when the q exceeds 0.1 e. Among fcc Cu planes, Cu(110) plane becomes the most stable surface when q = 0.2 e.

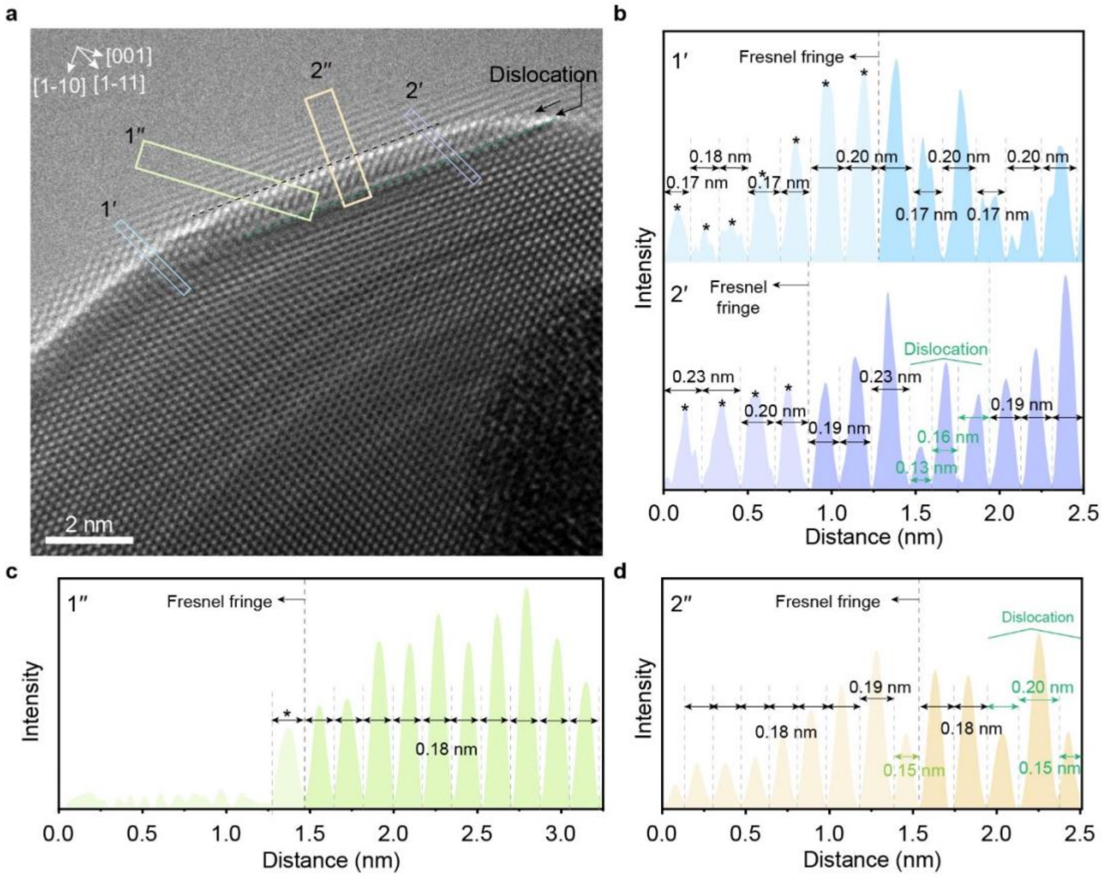

Supplementary Fig. 7 | Cu NP grown on the $[Gd_2C]^{2+}\cdot 2e^{-}$ electride after air exposure for 219 days. a, Enlarged HR-TEM image of the Cu NP presented in Fig. 2e of the manuscript. Dashed black lines and arrows indicate dislocations. **b**, Profiles for interatomic distance for the column in <111> direction from the boxed regions 1′ and 2′ marked in **a**, respectively. **c,d**, Profiles for interplanar distance from the boxed regions 1″ and 2″ marked in **a** for {002} and {111} planes, respectively. Vertical dashed lines indicate boundary of the Cu NP. Interatomic/interplanar distances vary due to the dislocations. However, the observed distances are shorter than 0.23 nm (interatomic distance) and 0.20 nm (interplanar distance), indicating no existence of Cu oxides that have larger distance than 0.23 nm (interatomic distance) and 0.20 nm (interplanar distance). Asterisk symbols correspond to the intensity obtained from the Fresnel fringe regions.

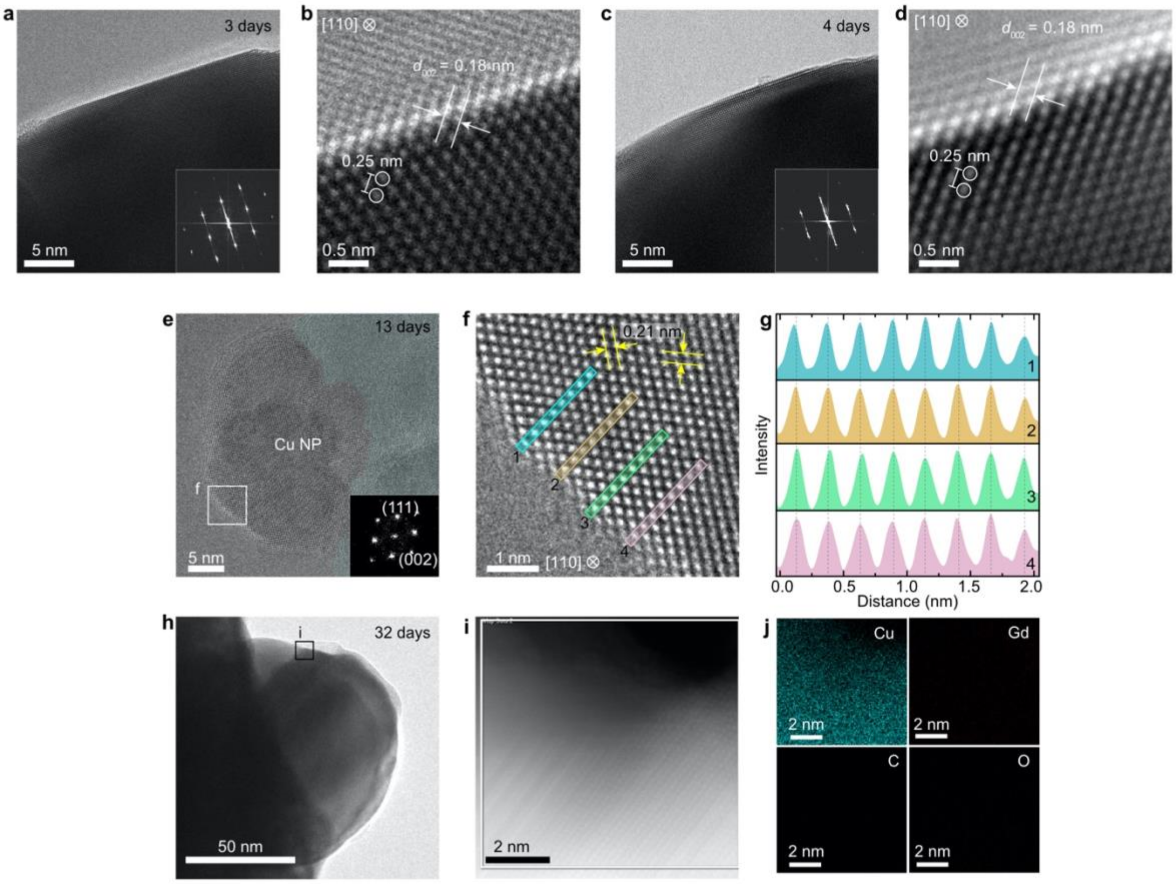

Supplementary Fig. 8 | Air-exposed Cu NPs grown on the $[Gd_2C]^{2+}\cdot 2e^{-}$ electride. a–d, HR-TEM images of the Cu NP in Supplementary Fig. 3a after air exposure for 3 and 4 days. No copper oxide was observed on the surface. **e**, TEM image of air-exposed Cu NP (13 days) measured with an acceleration voltage of 300 kV (inset: FFT pattern). **f**, Atomic arrangement on the surface region (boxed area in **e**). **g**, Interatomic distance profiles along the boxed arrays (1–4) in **f**, terminating at the outermost surface. **h–j**, EDX analysis was conducted on the surface (boxed region) of air-exposed negatively charged Cu NPs for 32 days (**h**), STEM-HAADF image of boxed region in **h** (**i**). Elemental mappings from copper, gadolinium, carbon, and oxygen (**j**).

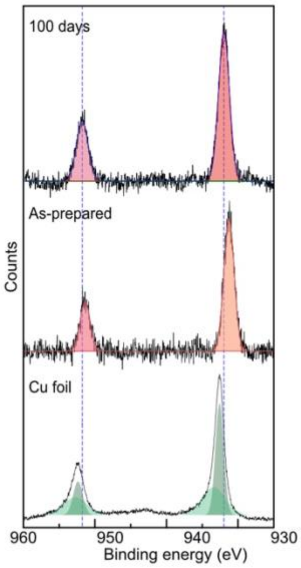

Supplementary Fig. 9 | Negatively charged surface state of air-exposed Cu NPs for 100 days. Binding energy for Cu 2$p_{3/2}$ of air-exposed Cu NPs (932.0 eV) for 100 days shows a shift toward lower energy compared to reference Cu metal (932.6 eV), reflecting the negatively charged state of Cu NPs. Although the shift degree was reduced from as-prepared Cu NPs (931.8 eV), it is clear that the Cu NPs maintain the surface with excess electrons after exposing to air for a long time.

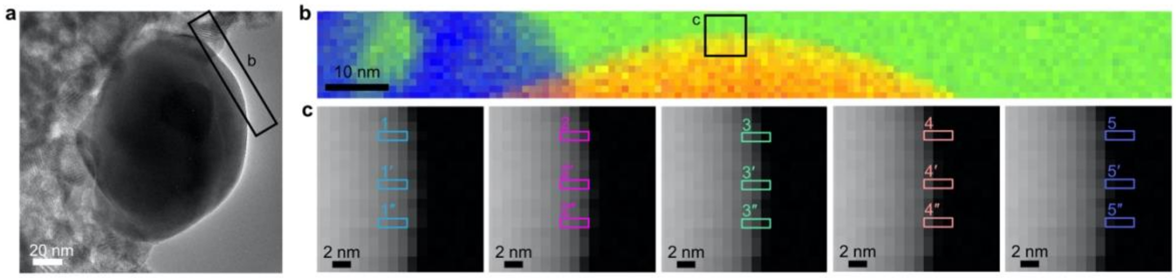

Supplementary Fig. 10 | EELS mapping of the non-oxidized Cu NPs. **a**, TEM image of the Cu NPs with the negatively charged surface state. **b**, STEM-EELS mapping image showing the distribution of Cu *L* edge (red), O *K* edge (blue) and the vacuum (green). **c**, STEM images with boxes indicating the pixels where the energy loss spectrum was collected and accumulated in Fig. 3c. Three pixels of 1 (1′ and 1″) and 2 (2′ and 2″) are of Cu NP. Three pixels of 5 (5′ and 5″) are of vacuum. 3 (3′ and 3″) and 4 (4′ and 4″) have two pixels and one pixel of Cu NP, respectively.

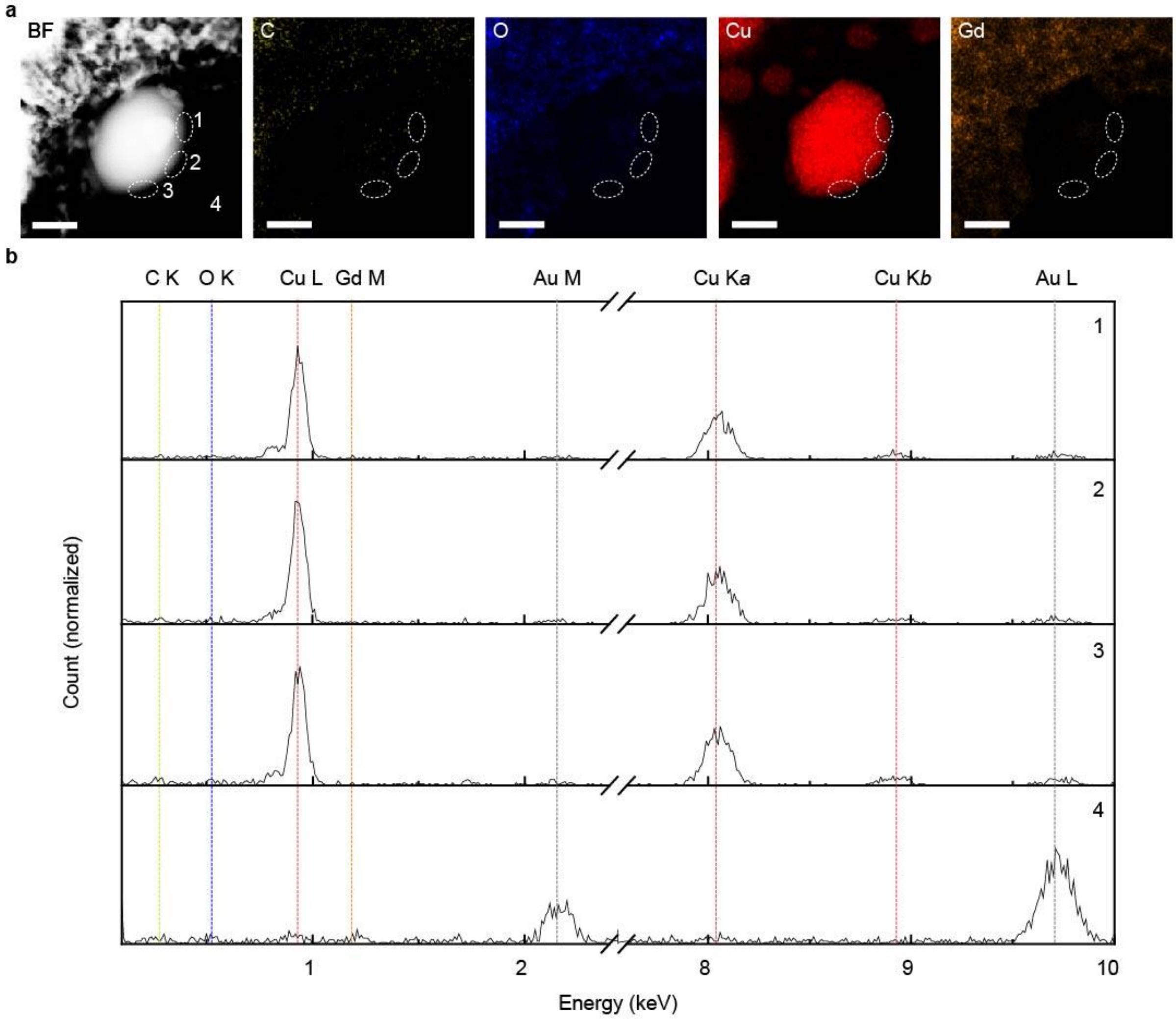

Supplementary Fig. 11 | EDS mapping of the non-oxidized Cu NPs. Elemental mapping results energy dispersive X-ray spectroscopy (EDS) (**a**) and spectra (**b**). Spectra 1, 2, 3 were taken from the surface regions of the Cu NP marked with dashed white circles, while spectrum 4 was obtained from the vacuum area. Scale bars correspond to 50 nm.

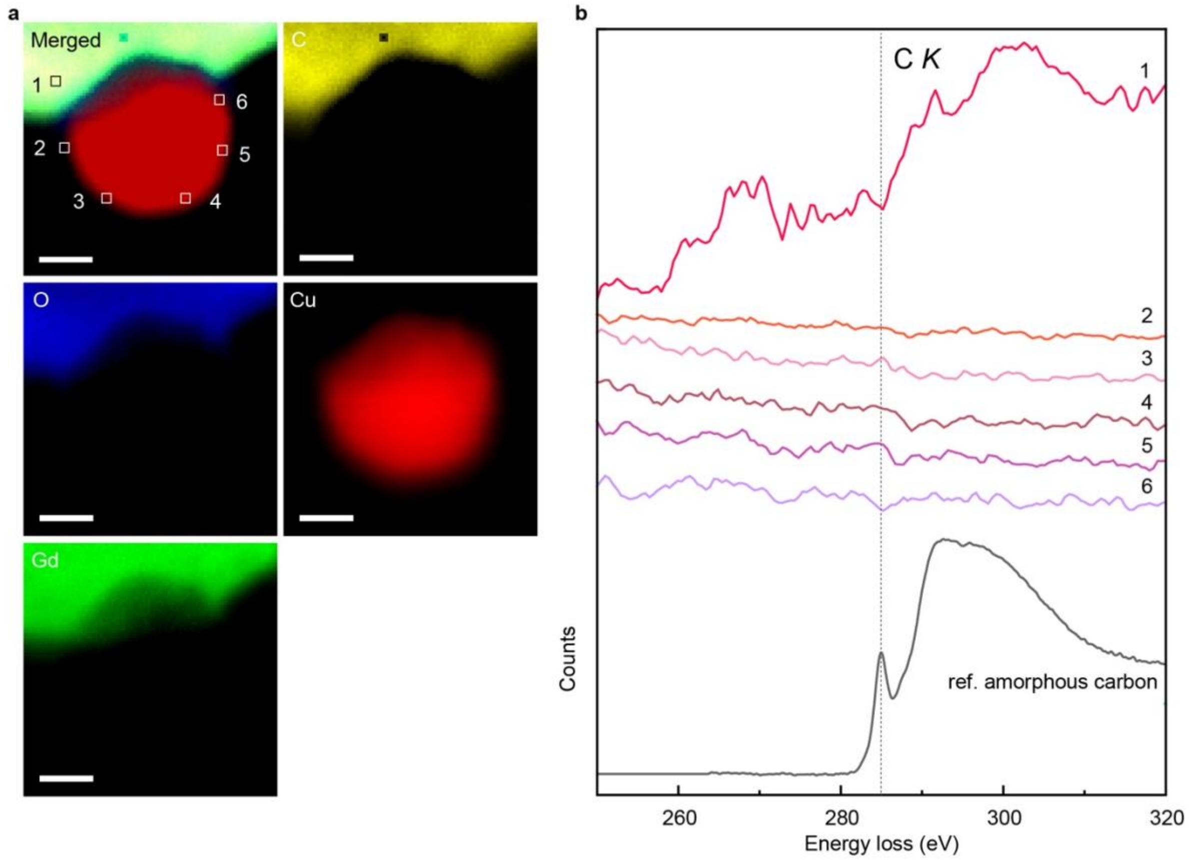

Supplementary Fig. 12 | EELS mapping of Cu NPs. **a**,**b**, EELS mapping images of the Cu NP grown on the $[Gd_2C]^{2+}\cdot 2e^-$ electride (air-exposed for 6 days) (**a**). ELNES of carbon *K* edge obtained from the surface regions marked with white circles in **a** (**b**). Scale bars correspond to 20 nm. Spectrum 1 was obtained from the oxidized $[Gd_2C]^{2+}\cdot 2e^-$ electride. Surface of Cu NP grown on the $[Gd_2C]^{2+}\cdot 2e^-$ electride (air-exposed for 6 days) doesn't exhibit any carbon adsorption (spectra 2–6).

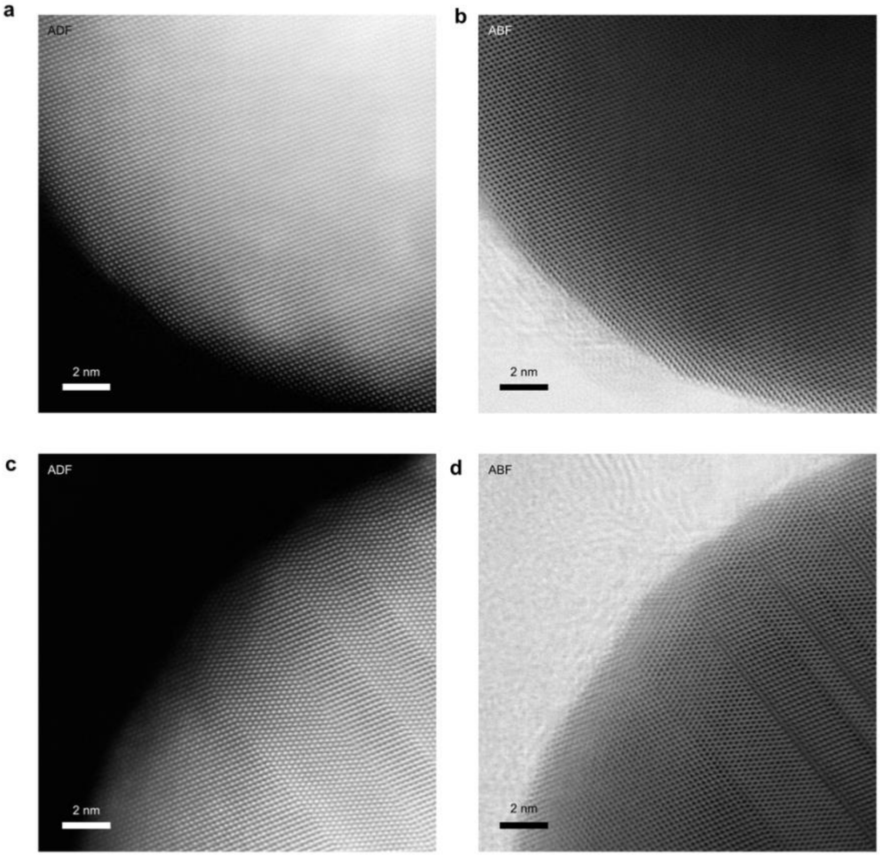

Supplementary Fig. 13 | ADF and ABF STEM images the non-oxidized Cu NPs. **a**,**b**, STEM images of the Cu NP (presented in Fig. 3d). **c**,**d**, STEM images showing another part of the same Cu NP. Both ADF and ABF-STEM images show bare and non-oxidized surface structure of the Cu NP.

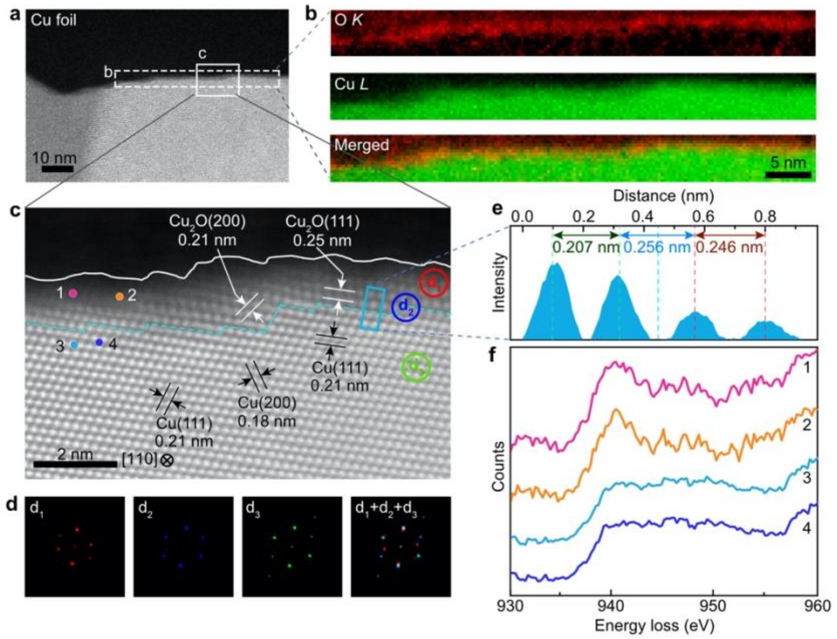

Supplementary Fig. 14 | EELS-STEM analysis on the reference Cu foil. a–**f**, EELS-STEM analysis on the commercial Cu foil. STEM image of Cu foil (**a**) and EELS mapping results (**b**). **c**, Atomic-scale ADF-STEM image of Cu foil. **d**, FFT patterns obtained at the marked regions in **c** (d_1: Cu_2O (red), d_2: mixed region of Cu and Cu_2O (blue), d_3: Cu metal (green)). Unmatched FFT pattern of $d_1 + d_2 + d_3$ clearly depicts the difference in the crystal structure of the surface and bulk of commercial Cu foil. **e**, Interplanar distance profile at the boundary for a boxed region in **c**. **f**, Corresponding energy loss spectrum of Cu L edge showing white line on the surface oxide region (positions 1 and 2).

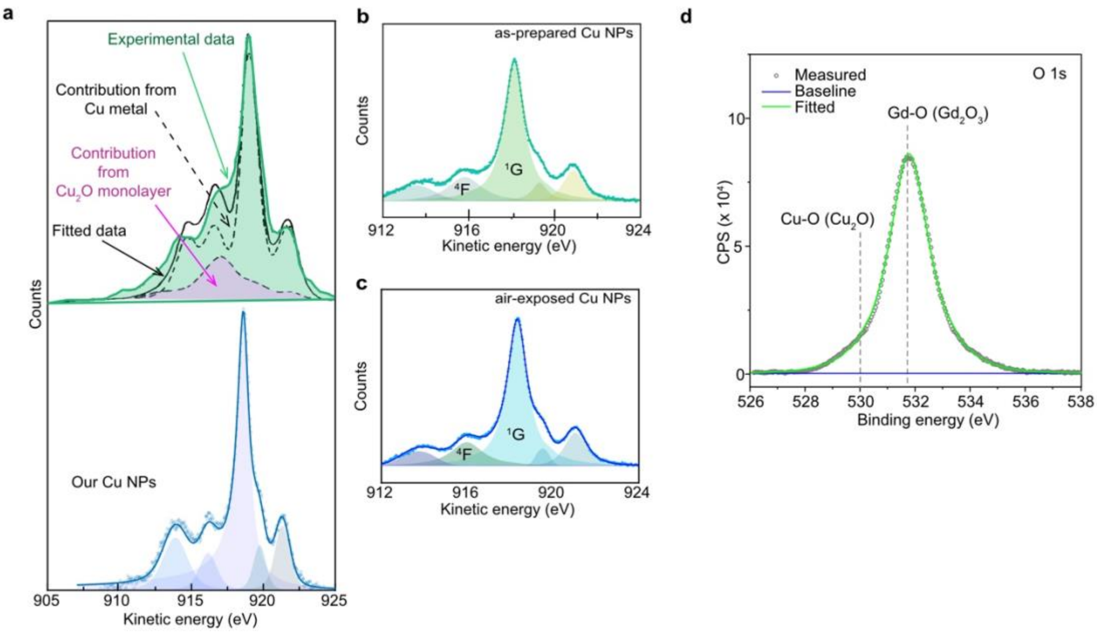

Supplementary Fig. 15 | Comparison of Auger electron spectra for our Cu NPs with the Cu metal disc and O 1s XPS. a, Cu Auger electron spectra of Cu disc with monolayer Cu_2O on the surface (top, adapted with permission from ref. 37) and Cu NPs with excess electrons (bottom), which is identical in Fig. 3**g**, except the background subtraction for comparison. **b**, **c**, AES of the as-prepared Cu NPs (**b**) and air-exposed Cu NPs for a day (**c**). As the position of the peak for ^4F state term (~918 eV) coincides with the most prominent peak for Cu oxides, it is necessary to compare the ratio of the ^4F to ^1G peak area from our samples with the literature value[35]. The relative peak area ratio between two peaks, obtained from the sputtered polycrystalline Cu with impurity below 1% was 0.24. In AES fitting results for our Cu NPs, both as-prepared and air-exposed samples show 0.24 for ^4F to ^1G peak area ratio. Thus, AES of both of our samples show the characteristics of non-oxidized Cu surface and the intensities of each peak are in a good agreement with the reported values. **d**, XPS of O 1*s* from the Cu NPs on the oxidized $[Gd_2C]^{2+}\cdot 2e^-$ electride. O 1*s* XPS spectrum showed the strong and symmetric feature of O 1s peak from a single peak. From the reported value[52], the strong peak of O 1s at 531.6 eV is originated from Gd_2O_3. The symmetric feature of O 1s peak by single peak fitting strongly indicates that there is no other oxygen species, including O 1s of the Cu_2O that exhibits a peak at 530.0 eV[37]. It is thus evident that the XPS O 1*s* spectrum from our sample proves no oxidation at the surface of Cu NPs on the $[Gd_2C]^{2+}\cdot 2e^-$ electride.

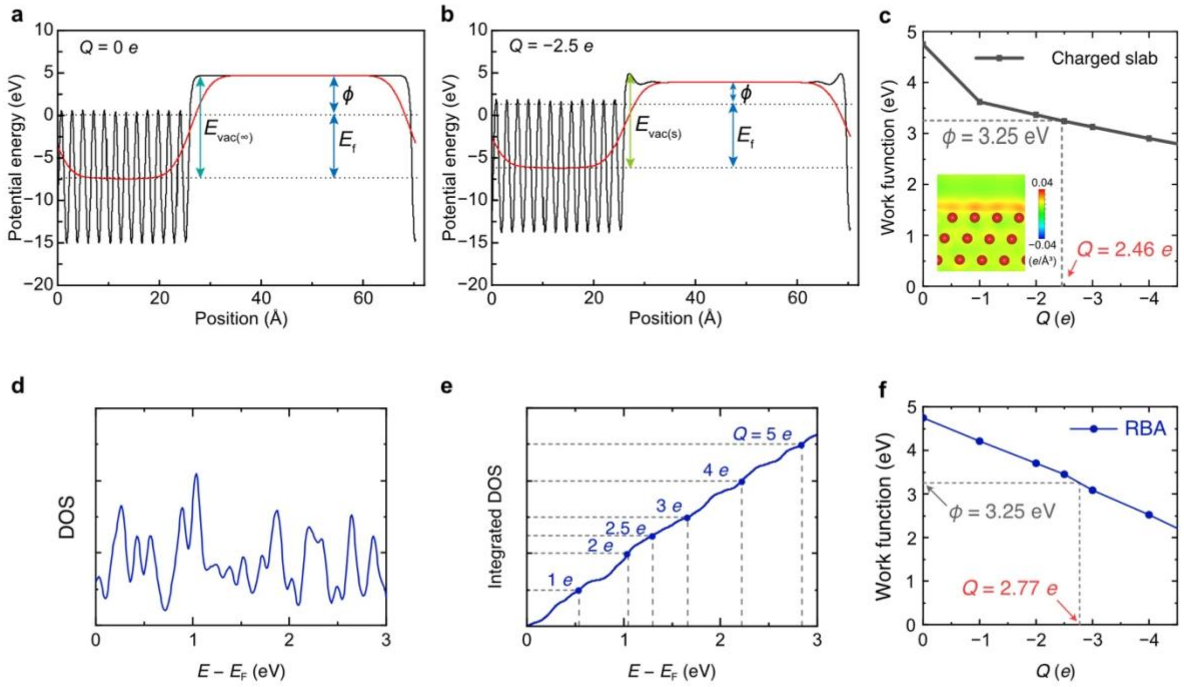

Supplementary Fig. 16 | Calculated work functions (ϕ) as a function of excess electrons (Q). a,**b**, Calculated potential energy for the neutral (Q = 0 e) (**a**) and the charged (Q = –2.5 e) (**b**) Cu(111) slabs as a function of position perpendicular to the surface of Cu(111) slab. Work function of Cu is defined by subtracting the electron energy at the metal surface, $E_{vac(s)}$, by the Fermi energy, E_f[53]. **c**, Calculated work functions as a function of Q obtained by the charged slab method(**a**,**b**). Determination of the excess charge density Q as −2.46 e for the present oxidation-resistant Cu NPs based on the experimentally obtained work functions of Cu by KPFM (average work function, ϕ_{av} ~3.2 eV) and STS (ϕ_{av} ~3.3 eV) measurements. Inset: charge density difference map of the flat Cu(111) surface with Q = −2.5 e showing the accumulation of excess electrons at the surface. **d**,**e**, Calculated density of states (DOS) (**d**) and integrated DOS (**e**) for the neutral (Q = 0 e) Cu(111) slab. Q values in **e** indicate the number of excess charges per surface Cu atom. **f**, Calculated work functions as a function of Q obtained by RBA method to check the validity of the work function values in **c**. For RBA method, the work function change of Cu (111) slab after adopting the excess electrons can be defined as $\Delta\phi = \phi_{excess} - (\phi_{neutral} - \Delta E_F)$, where ϕ_{excess} and $\phi_{neutral}$ are the work functions of the excess and neutral Cu slabs, respectively, and ΔE_F is the variation of the Fermi level of the system due to the excess electrons. The ΔE_F values are extracted by the integration of DOS above the Fermi level (**d**,**e**). The Q values obtained by RBA methods are –2.77 e as the work function of the Cu (111) slab is 3.25 eV, which value is close to that (2.46 e) obtained by charged slab method (**c**).

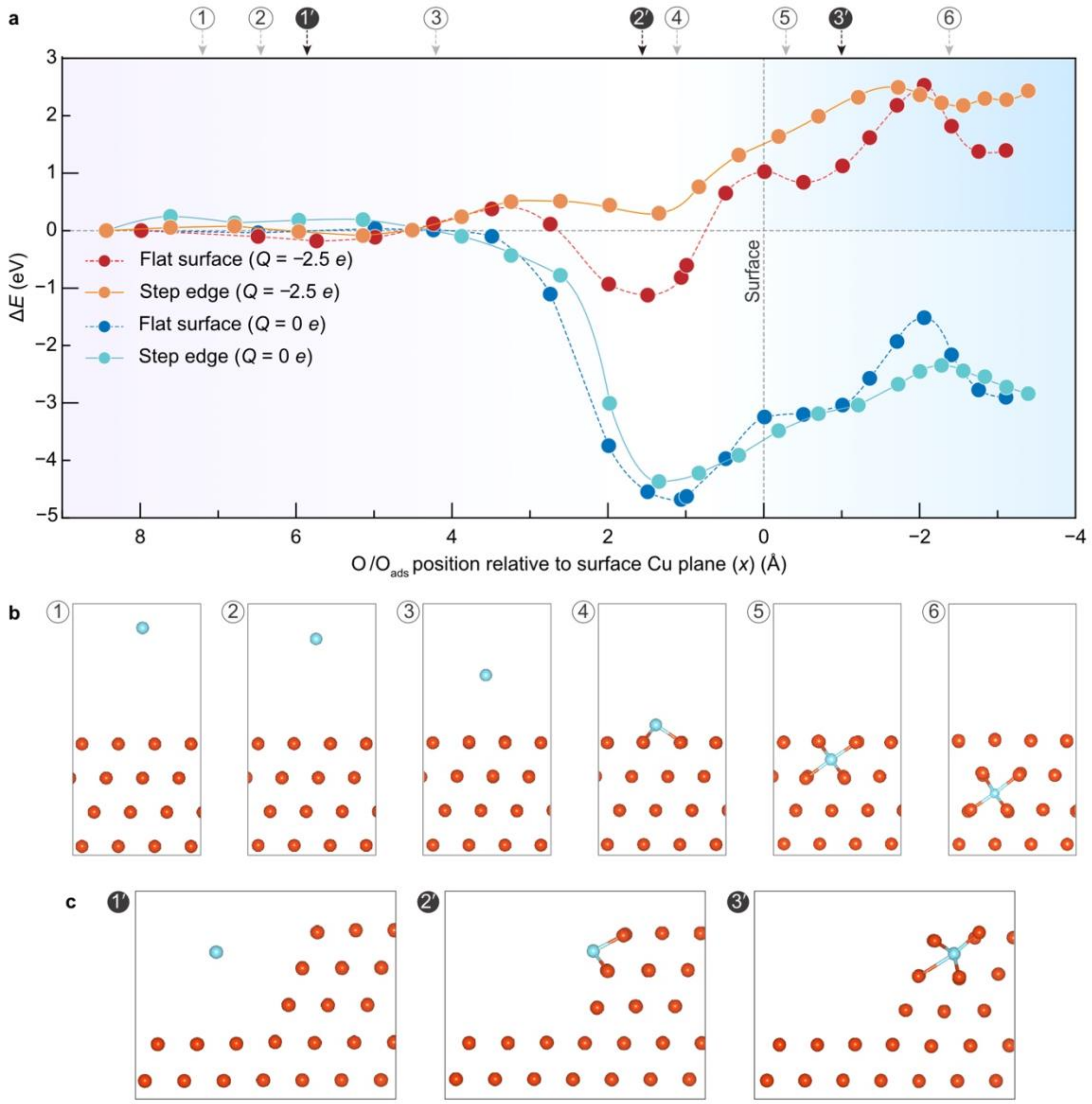

Supplementary Fig. 17 | Calculated energetics of oxidation process for the Cu with excess electrons. a–**c**, Oxidation process on the flat and stepped Cu lattice. Relative energy profile of the flat and stepped Cu lattices (**a**). Configurations of the oxidation process on the flat fcc Cu (111) lattice (**b**). 1–3: Oxygen approaches to the Cu surface, 4: chemisorbed oxygen, 5–6: Oxygen penetrates the Cu subsurface. Configurations of the oxidation process on the Cu lattice with 3-atomic step layer (**c**). 1′: Oxygen approaches to the stepped Cu surface, 2′: chemisorbed oxygen, 3′: Oxygen penetrates the Cu subsurface.

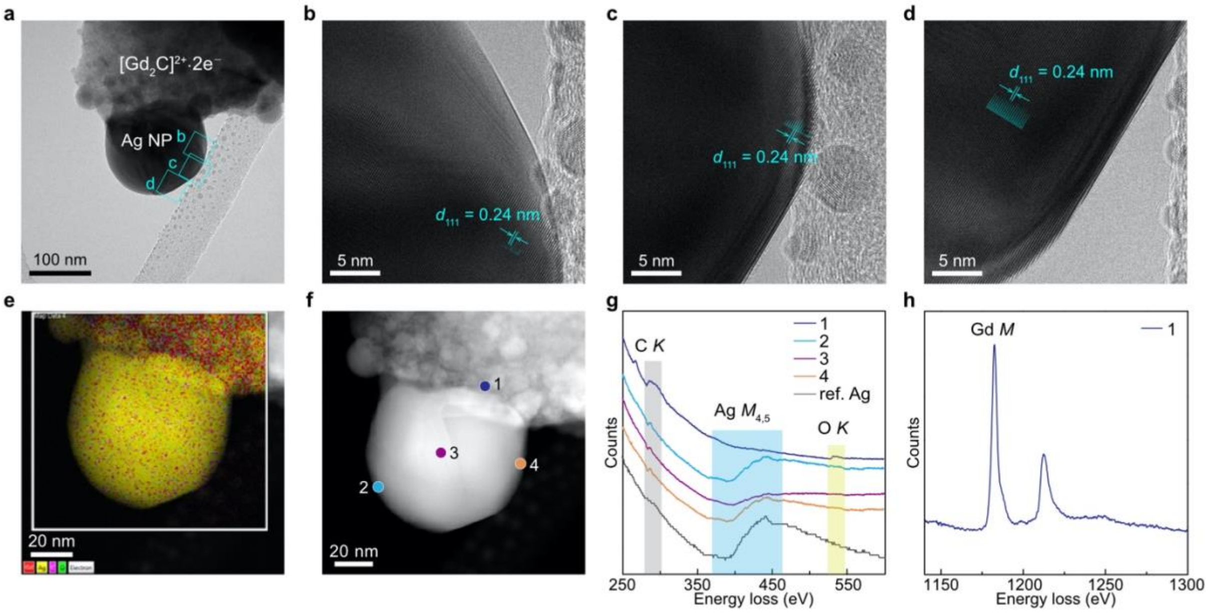

Supplementary Fig. 18 | Expansion of the synthetic methodology for non-oxidized Ag NPs. a–h, Ag NPs grown using $[Gd_2C]^{2+}\cdot 2e^-$ electride. TEM images showing the non-oxidized surface without any silver oxide moieties (**a–d**). HR-TEM images from the marked region in **a** with d-spacing profile corresponding to (111) planes of Ag (**b**, **c**, and **d**). **e**, EDX analysis of Ag NP of Ag (yellow), Gd (red), C (magenta), and O (green). EELS results obtained from the points marked with cyan circles in HAADF-STEM image (**f**), and EEL spectra showing C *K* edge (~284 eV), Ag *M* edge (364–460 eV), O *K* edge (532 eV) (**g**). As the spectrum from point 1 is located on oxidized $[Gd_2C]^{2+}\cdot 2e^-$ electride, the signal of Ag *M* edge is absent while the signal of O *K* edge is observed at near 532 eV. For the spectra acquired from points 2−4, distinct EELS bands at Ag *M* edge are collected. ELNES of Gd shows a white line of Gd with the oxidation state of 3+ (**h**).

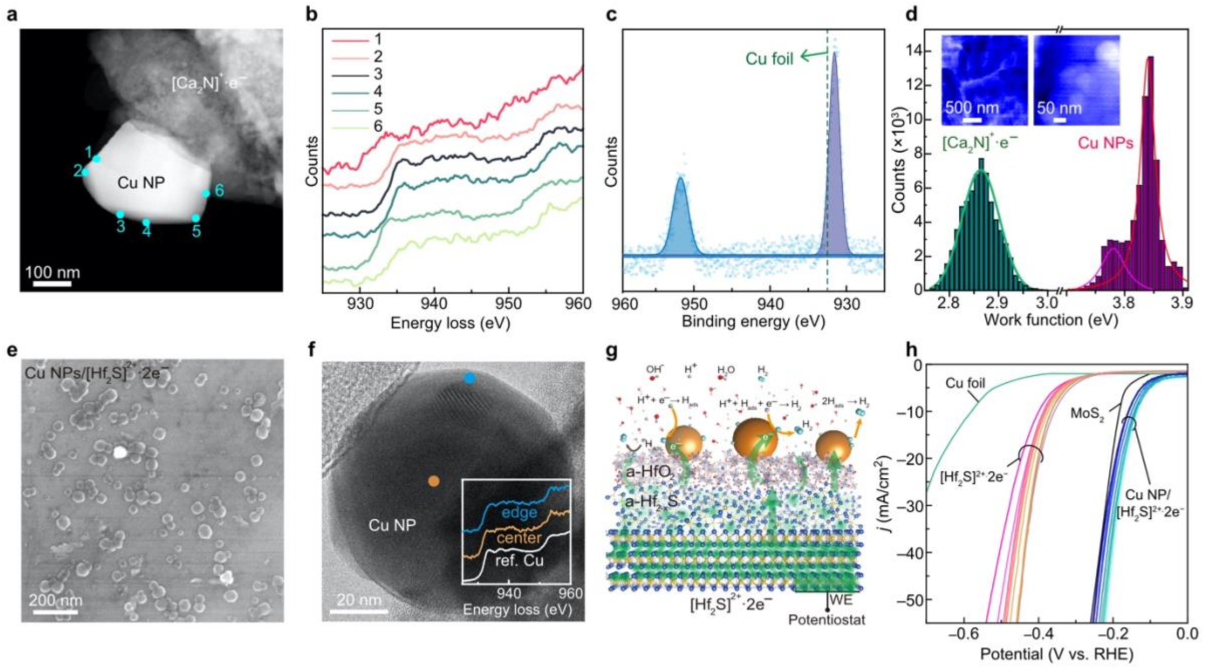

Supplementary Fig. 19 | Expansion of the synthetic methodology for non-oxidized Cu NPs using other electrides. a–d, Synthesis of Cu NPs using $[Ca_2N]^+ \cdot e^-$ electride[17]. HAADF-STEM image of Cu NPs grown on $[Ca_2N]^+ \cdot e^-$ electride (**a**) and EELS data without any white line from the marked regions in **a** (**b**). Cu 2*p* XPS of Cu NPs grown on $[Ca_2N]^+ \cdot e^-$ electride (**c**). Cu $2p_{3/2}$ peak is located at 931.56 eV, which shows the negative shift to lower binding energy compared to the Cu $2p_{3/2}$ of Cu foil (green dashed line). **d**, KPFM measurements of $[Ca_2N]^+ \cdot e^-$ electride (green bars) and from Cu NPs on $[Ca_2N]^+ \cdot e^-$ electride (purple bars). Average work function values of $[Ca_2N]^+ \cdot e^-$ electride is ~2.87 eV which is similar with the measured value by UPS (2.6 eV)[13]. The work function value of Cu NPs was ~3.84 eV, which is a distinctively lower value than that (4.5 eV) of Cu metal. These results validate the effective transfer of excess electrons from electrides with low work function values to Cu NPs, enabling the negatively charged state (Insets: CPD mapping images of $[Ca_2N]^+ \cdot e^-$ electride (left) and Cu NPs grown on $[Ca_2N]^+ \cdot e^-$ electride (right)). **e–h**, Synthesis of Cu NPs using $[Hf_2S]^{2+} \cdot 2e^-$ electride[54] and application in electrocatalytic hydrogen evolution reaction (HER). SEM image of Cu NPs on $[Hf_2S]^{2+} \cdot 2e^-$ electride (Cu NPs/$[Hf_2S]^{2+} \cdot 2e^-$) (**e**). TEM image of a Cu NP (**f**) and EEL spectra of Cu obtained from the edge (blue) and centre (yellow) (inset). Structural model and possible pathway for HER over Cu NP/$[Hf_2S]^{2+} \cdot 2e^-$ electride (**g**) and HER performance of Cu foil[55], $[Hf_2S]^{2+} \cdot 2e^-$ electride, defect-rich ultrathin nanosheet MoS_2, and $[Hf_2S]^{2+} \cdot 2e^-$ electride with Cu NPs for 5,000 cycles (**h**). (Schematic illustration of $[Hf_2S]^{2+} \cdot 2e^-$ electride and linear sweep voltammetry (LSV) curves of $[Hf_2S]^{2+} \cdot 2e^-$ electride are adapted with permission from ref. 54,

© The Authors, some rights reserved; exclusive licensee AAAS. Distributed under a CC BY-NC 4.0 License (http://creativecommons.org/licenses/by-nc/4.0/)). LSV curves were measured in 0.5 M sulfuric acid using a saturated calomel electrode as a reference electrode and a graphite rod as a counter electrode (scan rate: 5 mV/s). Chemical stability of $[Hf_2S]^{2+}\cdot 2e^-$ in air, water and an acidic environment is originated from the self-passivation layer at the surface, which is composed of the amorphous HfO_2 (a-HfO_2) and amorphous $Hf_{2-x}S$ (a-$Hf_{2-x}S$) on the crystalline $[Hf_2S]^{2+}\cdot 2e^-$. Interestingly, moderate HER catalytic activity of $[Hf_2S]^{2+}\cdot 2e^-$ electride is improved by Cu NPs, showing similar activity as defect-rich ultrathin nanosheet MoS_2 and no decrease in the performance during 5,000 cycles.

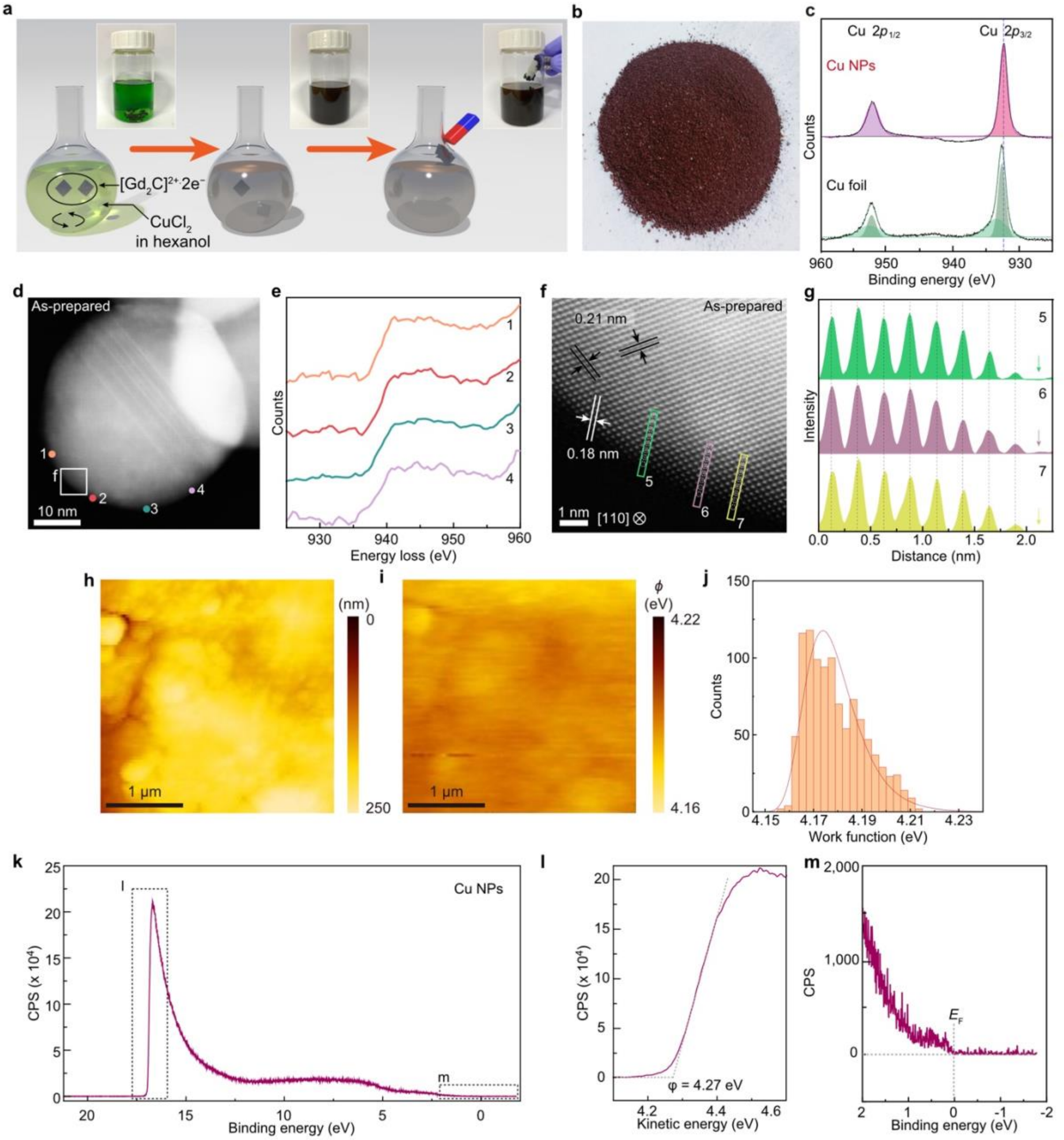

Supplementary Fig. 20 | Mass production of the non-oxidized Cu NPs via a solution process. a, Schematic illustration showing wet chemical synthesis route and separation for Cu NPs and photographs showing a colour transition of the solution with respect to the reaction time. **b**, Photograph of the obtained Cu NP powder. **c**, Cu 2*p* XPS spectra of the wet chemically synthesized Cu NPs (top) and reference Cu foil (bottom) (**c**). Cu $2p_{3/2}$ peak of the Cu NPs shows negatively shifted binding energy (932.2 eV) compared to the peak of Cu foil (932.6 eV). **d**–**g**, STEM-EELS analysis of as-prepared Cu NPs. STEM images of a Cu NP (**d**) and corresponding EEL spectra obtained at the points in STEM images (**e**). Enlarged HR-STEM images (**f**) and interatomic distance profiles from the boxed region in **f** indicate the non-oxidized surface of the solution-processed Cu NPs (**g**). **h**–**j**, KPFM measurement of the Cu NPs

by wet chemical synthesis. Topography (**h**) and work function mapping image (CPD values were converted to work function by calibrating work function of the tip with HOPG) (**i**) with the histogram of the obtained work function values (~4.2 eV in average) (**j**) imply the negatively charged surface state of the solution-processed Cu NPs. k–m, UPS measurement of the Cu NPs by wet chemical synthesis. **k**, UPS spectrum of our Cu NPs prepared by solution process, measured with photon energy of 21.218 eV. **l**, Enlarged cut-off region of UPS spectrum in kinetic energy giving work function of Cu NPs as 4.27 eV. **m**, Enlarged Fermi edge region in binding energy.

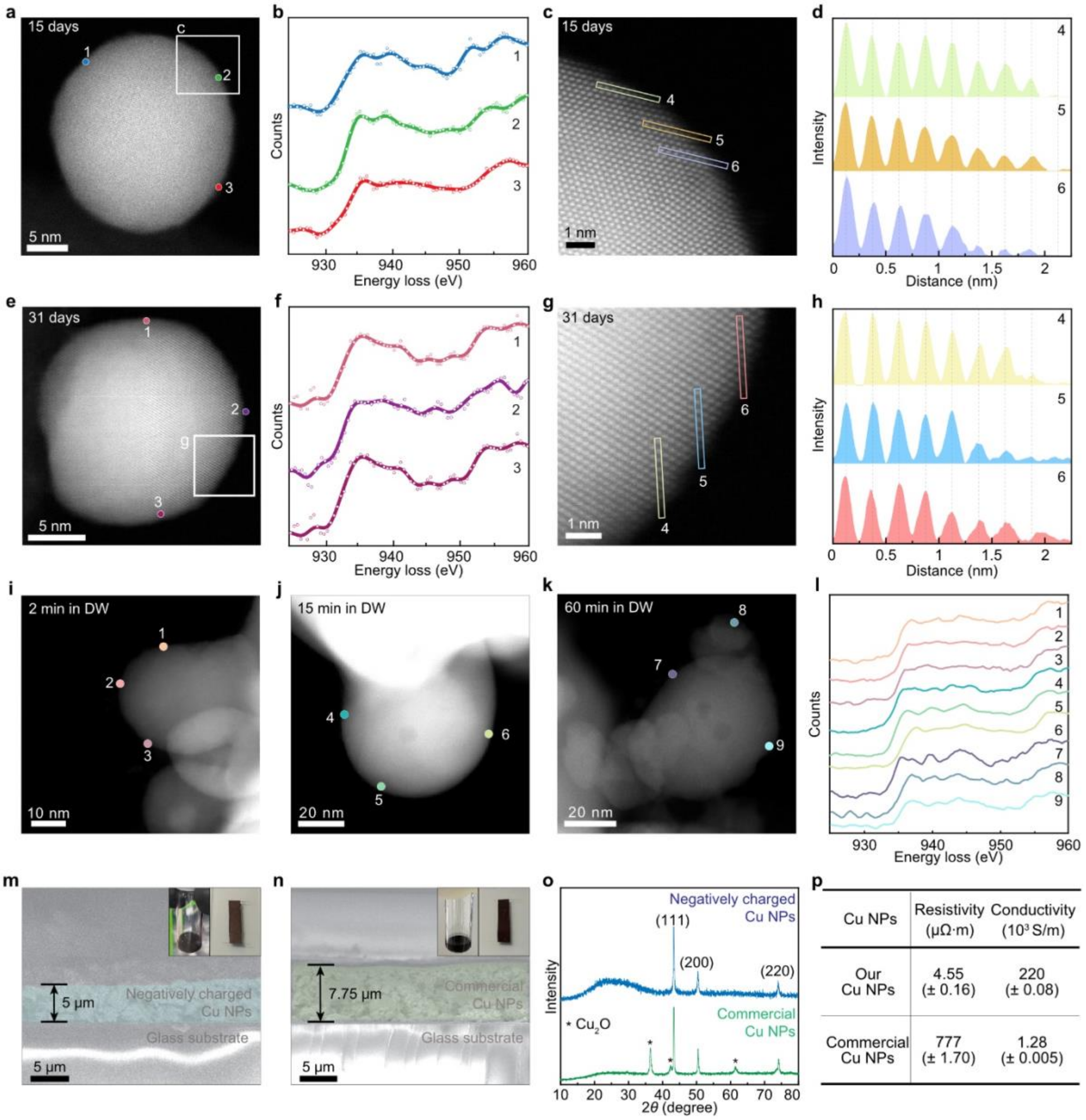

Supplementary Fig. 21 | Stability and application of the solution-processed Cu NPs. a–h, STEM-EELS analysis of the solution-processed Cu NPs. Cu NPs were analyzed after 15 days (**a**–**d**) and 31 days (**e**–**h**) in air, respectively. STEM images of a Cu NP (**a**,**e**) and corresponding EEL spectra obtained at the points in STEM images (**b**,**f**). Enlarged HR-STEM images (**c**,**g**) and interatomic distance profiles from the boxed region in **c**,**g** indicate the non-oxidized surface of the solution-processed Cu NPs (**d**,**h**). **i**–**l**, Water stability of the solution-processed Cu NPs. STEM images of water-immersed Cu NPs for 2 min (**i**), 15 min (**j**), and 60 min (**k**) in deionized water and corresponding Cu L edge EEL spectra (**l**) obtained at the points in STEM images display that the surfaces of water-immersed Cu NPs preserve the metallic state. **m**–**p**, Fabrication of the electrodes using Cu NP inks. Cross-sectional SEM images of the electrodes by using wet chemically synthesized Cu NPs (**q**) and commercial Cu NPs (**r**). Insets:

photograph of the Cu NP inks prepared with wet chemically synthesized Cu NPs (**m**) and commercial Cu NPs (**n**) and prepared electrodes. **o**, XRD patterns of the air-exposed electrode using wet chemically synthesized Cu NPs (top) and commercial Cu NPs (bottom). **p**, Data set of electrical properties of the electrodes prepared using inks of negatively charged Cu NPs and commercial Cu NPs with polyvinylpyrrolidone (PVP) and isopropyl alcohol (IPA). Note that the electrodes with our negatively charged bare Cu NPs exhibit a constant electrical conductivity, even after 10 days air exposure, indicating that our Cu NPs in the electrodes maintain their metallic state without surface oxidation.

References

52. Majeed, S. & Shivashankar, S. A. Rapid, microwave-assisted synthesis of Gd_2O_3 and Eu:Gd_2O_3 nanocrystals: characterization, magnetic, optical and biological studies. *J. Mater. Chem. B* **2**, 5585–5593 (2014).

53. Hölzl, J., Schulte, F. K. & Wagner, H. *Solid surface physics*. (Springer, 2006).

54. Kang, S. H. *et al.* Water- and acid-stable self-passivated dihafnium sulfide electride and its persistent electrocatalytic reaction. *Sci. Adv.* **6**, eaba7416 (2020).

55. Raoof, J.-B., Ojani, R., Kiani, A. & Rashid-Nadimi, S. Fabrication of highly porous Pt coated nanostructured Cu-foam modified copper electrode and its enhanced catalytic ability for hydrogen evolution reaction. *Int. J. Hydrog. Energy* **35**, 452-458 (2010).
